# Liver damage favors the eliminations of HBV integration and clonal hepatocytes in chronic hepatitis B

**DOI:** 10.1007/s12072-020-10125-y

**Published:** 2021-02-03

**Authors:** Gang Hu, Ming X. Huang, Wei Y. Li, Chong J. Gan, Wen X. Dong, Xiao M. Peng

**Affiliations:** 1 Center of Infectious Diseases, the Fifth Affiliated Hospital, Sun Yat-Sen University, 52 Meihua East Road, Zhuhai, 519000 Guangdong China; 2 Jining Medical University, Jining, 272057 Shandong China

**Keywords:** Hepatitis B virus, Chronic hepatitis B, Immune clearance, Liver damage, Viral DNA integration, Clonal expansion, Hepatocellular carcinoma, Hepatocarcinogenesis, Liver biopsy, Inflammation activity, Next-generation DNA sequencing, High-throughput viral integration detection

## Abstract

**Background:**

HBV integration is suspected to be an obstinate risk factor for hepatocellular carcinoma (HCC) in the era of antiviral therapy. Integration events start to occur in the immunotolerance phase, but their fates in the immune clearance phase have not yet been clarified. Here, we report the influences of liver damage on HBV integration and clonal hepatocyte expansion in patients with chronic hepatitis B (CHB).

**Methods:**

HBV integration breakpoints in liver biopsy samples from 54 CHB patients were detected using a modified next-generation sequencing assay.

**Results:**

A total of 3729 (69 per sample) integration breakpoints were found in the human genome, including some hotspot genes and KEGG pathways, especially in patients with abnormal transaminases. The number of breakpoint types, an integration risk parameter, was negatively correlated with HBV DNA load and transaminase levels. The average, maximum and total frequencies of given breakpoint types, parameters of clonal hepatocyte expansion, were negatively correlated with HBV DNA load, transaminase levels and liver inflammation activity grade score. The HBV DNA load and inflammation activity grade score were further found to be positively correlated with transaminase levels. Moreover, nucleos(t)ide analog (NUC) treatment that normalized transaminases nonsignificantly reduced the types, but significantly increased the average frequency and negated the enrichments of integration breakpoints.

**Conclusion:**

Liver damage mainly removed the inventories of viral integration and clonal hepatocytes in CHB. NUC treatment may have reduced HBV integration but clearly increased clonal hepatocyte expansion, which may explain why HCC risk cannot be ruled out by NUC treatment.

**Supplementary Information:**

The online version contains supplementary material available at 10.1007/s12072-020-10125-y.

## Introduction

Hepatitis B virus (HBV) infection is the major cause of hepatocellular carcinoma (HCC) regardless of current antiviral therapy with nucleos(t)ide analogs (NUCs). The burden of liver cancer is increasing [1], and the risk of HBV-related HCC, despite virological and biochemical responses, is still high in the first five years [2], steadily persists over time [3], and remains stable, particularly in patients with cirrhosis [4] or is at least not eliminated [5]. The HCC risk still exists among those patients outside current treatment criteria or even exists in individuals with HBV that has been functionally cleared [6, 7]. These above findings suggest that there are some obstinate HCC-driving factors other than viral replication and hepatitis.

HBV integration, oncoprotein hepatitis B x protein, chronic necroinflammation and hepatocellular regeneration, together with chemical carcinogenesis, generally account for hepatocarcinogenesis [8]. Among these factors, HBV integration has the potential to be one of those obstinate driving factors. It can lead to chromosomal instability, genomic damage and residual expression of viral oncoproteins, which are all known as selective advantages for tumor progression [9]. Indeed, HBV integration has been found in more than 80% of HBV-related HCC cases, all tumor single cells exhibit the same HBV integration in monoclonal HCC, and integration sites or integration-related genes are believed to be important for hepatocarcinogenesis [10, 11]. HBV integration events randomly occur as early as in the immunotolerance phase of patients [12] or 1–3 h post infection in model cells [13]. Viral integration, thus, has enough time to play roles in narrowing the hepatocyte population by clonal hepatocyte expansion, similar to other HCC contributors [14]. Clonal hepatocyte expansion, a major risk factor for HCC [15], often occurs in the later stages of infection such as cirrhosis. It is estimated that at least half of cirrhotic nodules are clonal [16]. However, clonal hepatocyte expansion also occurs as early as in the liver of patients with so-called immunotolerance [12, 17]. Therefore, the subsequent fates and evolution of early viral integration in the immune clearance phase (chronic hepatitis) are important parts of hepatocarcinogenesis. Unfortunately, only very few studies have focused on viral integration events in chronic hepatitis B (CHB), and none of them have discussed the subsequent fates and evolution to date.

In this study, HBV integration was successfully detected using a high-throughput viral integration detection (HIVID) assay in liver biopsy samples from 54 CHB patients. The subsequent fates of HBV integrations and clonal hepatocytes in the stage of CHB were mainly influenced by liver damage surrogate indicators, alanine aminotransferase (ALT), aspartate aminotransferase (AST) and liver inflammation activity grade score. NUC treatment that remitted liver damage was preliminarily found to perhaps reduce viral integration but undoubtedly increase clonal hepatocytes, which may explain why HCC risk cannot be ruled out by NUC treatment.

## Materials and methods

### Subjects

A total of 54 CHB patients were enrolled in this study at the Fifth Affiliated Hospital of Sun Yat-sen University from January 2018 to September 2019.The clinical data of these patients are shown in Table S1. Among these patients, 2 and 11 patients received NUC treatment for less and more than 3 months, respectively. For convenience, they were further divided into NUC-treated (11 patients treated with NUC for more than 3 months), NUC-untreated (41 patients without NUC treatment) and non-NUC-treated (41 untreated and 2 temporally treated patients) groups as showen in Fig. S1. The general information about antiviral treatment is shown in Table S2. 

### Samples, DNA extraction, HBV DNA capture and high-throughput sequencing

Liver biopsy samples from 54 CHB patients were collected and stored at – 80 °C until use. Genomic DNA was extracted according to the guidance of the Tiangen kit (Nanjing, China). The capture probes were designed according to the DNA sequences of 8 genotypes of HBV and synthesized by MyGenostics. The extracted DNA (1 μg) from each sample was broken into fragments of 150–200 bp in length by Covaris M220 (Covaris Inc., Woburn, MA). These fragments were purified, end blunted, ‘A’ tailed and adaptor ligated to obtain DNA library. The capture of HBV DNA in the library and DNA sequencing were conducted as previously reported [18].

### Detection of HBV integration breakpoints

HBV integration breakpoints were determined using the HIVID method as reported [18]. The breakpoints with support reads ≥ 10 were retained. ANNOVAR was used to annotate the integration breakpoints [19]. Because the integrated viral genome has been considered a strong cis-activator of the flanking genes and cis-acting enhancers influence their target genes over long distances [20], nearby genes of the breakpoints located in the intergenic region were included to calculate the affected gene frequency in HBV-integrated samples [21].

### Estimation of clonal hepatocyte expansion

HIVID primarily showed breakpoints with unique DNA sequences of human and viral genomes. Here, they were called the types of integration breakpoints. HIVID also provided the support reads that could be used to calculate the frequency (ectypal breakpoints) of each type of breakpoint in a given sample: given breakpoint type frequency = (total support reads/clean data bases (Mb))*150 (bp, read-length). Similar to virus–cell DNA junctions [17], breakpoints can also be used to estimate clonal hepatocyte expansion since identical integration breakpoints are assumed to originate from a single hepatocyte clone. Thus, the frequency of a given breakpoint represented the clone size of the same breakpoint-carrying hepatocytes. The clonal hepatocyte expansion of a given patient was estimated using these parameters of the average (the average frequency of all breakpoint types), maximum (the highest frequency among all breakpoint types) and total frequencies (the summation of frequencies of all breakpoint types) of the breakpoint types in 1 μg of genomic DNA extracted from the liver biopsy sample.

### Pathway analysis

Pathway enrichment analysis of the integration breakpoints based on Kyoto Encyclopedia of Genes and Genomes (KEGG) was conducted using Clusterprofiler software, an intelligent bioinformatic tool that also performs statistical and network analyses [22]. The significance threshold for altered biological processes/pathways was set at a corrected hypergeometric *p*-value of 0.05.

### Statistical analysis

Continuous variables with a normal distribution are represented by the mean and standard deviation (SD) and were compared using Student’s *t* test and Pearson correlation analysis. Continuous variables with a non-normal distribution are expressed as the median and interquartile range (IQR) and were compared using the Mann–Whitney *U* test and Spearman correlation analysis. The classification variables are expressed as numbers (%) and were compared by the *χ*^2^ test or Fisher’s exact test. *p* < 0.05 was considered statistically significant. All data were analyzed using SPSS (version 23.0) software.

## Results

### Characteristics of HBV integration

**Fig. 1:**
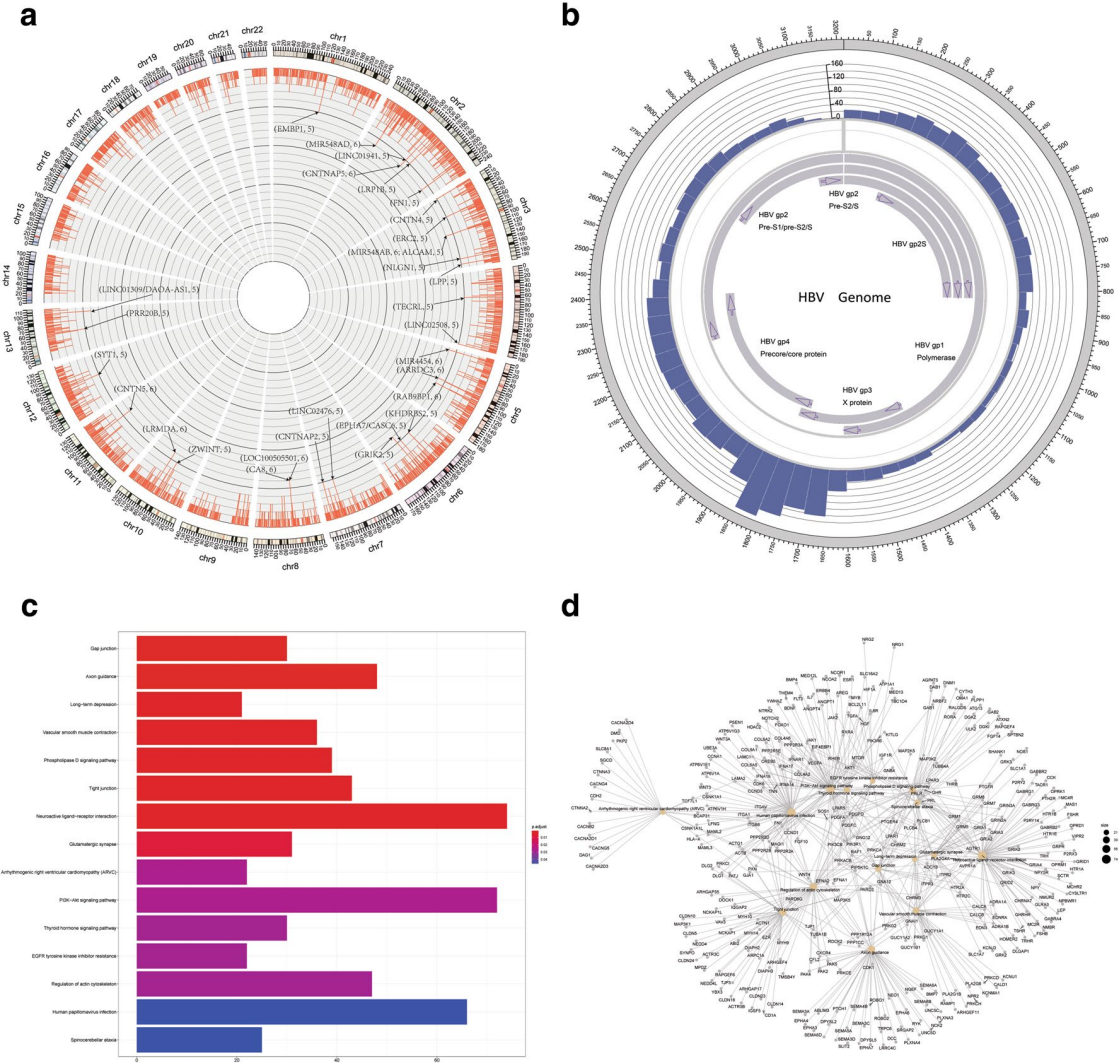
Distributions and enrichments of integration breakpoints in human and HBV genomes in 54 CHB patients. **a** Distribution in human genome. Each red bar represents the sample frequency of HBV integration breakpoints at a particular locus in the human genome (hg19). Histogram axis units represent number of samples. Some loci with a high frequency of integration are marked. **b** Distribution in HBV genome. Each blue bar represents the number of HBV integration breakpoints. **c** Histogram of the functional enrichments of HBV integration breakpoints in KEGG pathways. The vertical and horizontal axes illustrate the name of the pathway and the count of the enriched genes, respectively. The color shows statistical significance, with red relating to a smaller *p*-value. **d** Network map of HBV integration-related genes and pathways. The gray node in the figure represents a gene; the orange node represents the pathway, and the circle size represents the gene numbers in the pathway; the gray line between the nodes represents the interaction of genes

A total of 3729 (69 per sample) integration breakpoints were found in the human genomes of 54 CHB patients. There was a series of recurrent integration-related host genes (Fig. 1a). The breakpoint hotspots in the HBV genome were located in the region of 1 600–1 900 bp (Fig. 1b). KEGG pathway analysis revealed that several important human pathways are related to viral integration, including gap junction, tight junction, axon guidance, long-term depression, glutamatergic synapse, arrhythmogenic right ventricular cardiomyopathy, PI3K–Akt signaling, thyroid hormone signaling and EGFR tyrosine kinase inhibitor resistance (Fig. 1c). Network analysis showed that the three pathways of glutamatergic synapse, gap junction and long-term depression had stronger interaction effects among pathways (Fig. 1d).

### Correlation analyses of breakpoint types and frequencies with major clinical data

The landscape of viral integration and major clinical characteristics are shown in Fig. S2. The number of breakpoint types and the average, maximum and total frequencies of given breakpoint types were not significantly different when patients were stratified by age, sex, viral genotype, hepatitis B surface antigen (HBsAg) persistence, albumin (ALB) and alpha fetoprotein (AFP) (Table 1). However, patients with abnormal AST levels had significantly fewer breakpoint types, those with positive hepatitis B e antigen (HBeAg) had significantly lower average and maximum breakpoint frequencies, and those with a high load of serum HBV DNA (> 10,000 IU/mL), abnormal ALT or abnormal AST levels had significantly lower average, maximum and total breakpoint frequencies (Table 1). Furthermore, the types and frequencies of HBV integration breakpoints were correlated with clinical data in different manners (Fig. S3). The number of breakpoint types was positively correlated with the time of HBsAg persistence, but the average, maximum and total breakpoint frequencies were all negatively correlated with ALT, AST, HBV DNA load and HBeAg (except for total breakpoint frequency) (Fig. S3).

**Table 1:** HBV integration and major clinical data in chronic hepatitis B patients

Variables	Cases (*n*)	Breakpoint types (*n*)	Breakpoint frequencies (mean ± SD)		
			Average	Maximum	Total
Age (years)					
≤ 35	27	70.4 ± 96.9	27.8 ± 39.6	193.7 ± 347.5	1591.9 ± 2315.0
> 35	27	67.7 ± 75.6	27.5 ± 31.7	284.3 ± 373.1	2144.0 ± 2869.9
Gender					
Male	39	71.3 ± 89.3	29.2 ± 39.8	274.1 ± 391.9	1944.0 ± 2281.5
Female	15	63.2 ± 79.7	12.8 ± 17.5	147.7 ± 248.0	1670.3 ± 3369.8
HBV genotypes					
B	16	81.4 ± 80.5	24.1 ± 34.9	194.6 ± 264.4	2479.8 ± 3653.5
B/C	28	68.3 ± 101.3	29.1 ± 40.9	281.1 ± 430.5	1901.8 ± 2191.9
C	10	51.4 ± 37.4	12.9 ± 14.6	192.2 ± 281.9	794.4 ± 1059.2
HBsAg ( +) (years)					
≤ 15	31	65.9 ± 101.4	25.8 ± 37.9	232.5 ± 336.3	1949.5 ± 2754.3
> 15	23	73.3 ± 61.5	23.1 ± 33.1	247.7 ± 397.4	1758.0 ± 2426.5
HBsAg (IU/mL)					
≤ 1000	11	61.8 ± 72.8	33.1 ± 33.3	342.6 ± 463.9	2044.4 ± 2279.4
> 1000	43	70.9 ± 89.8	22.5 ± 36.3	212.5 ± 329.9	1822.9 ± 2695.7
HBeAg					
(+)	33	80.2 ± 106.1	13.2 ± 19.0*	119.1 ± 195.0*	1537.8 ± 2659.5
(-)	21	52.9 ± 40.3	41.3 ± 46.7	413.4 ± 466.1	2348.2 ± 2486.0
HBV DNA (IU/mL)					
≤ 10,000	21	72.6 ± 68.4	36.9 ± 34.1**	379.9 ± 399.2**	2901.2 ± 3004.2*
> 10,000	33	66.8 ± 96.6	16.9 ± 34.9	149.3 ± 306.2	1210.5 ± 2094.6
ALT (IU/L)					
≤ 40	22	73.9 ± 69.6	34.6 ± 31.8*	383.1 ± 376.1**	2825.1 ± 3141.8*
> 40	32	65.7 ± 97.7	17.8 ± 36.9	139.9 ± 317.7	1209.9 ± 1936.9
AST (IU/L)					
≤ 40	31	86.8 ± 96.1**	32.1 ± 30.5**	340.2 ± 359.3**	2797.9 ± 2926.5*
> 40	23	45.2 ± 64.9	14.6 ± 40.1	102.6 ± 319.9	614.6 ± 1309.4
ALB (g/L)					
≤ 40	8	39.1 ± 59.6	18.5 ± 40.8	128.5 ± 268.0	817.6 ± 1415.2
> 40	46	74.3 ± 89.4	25.7 ± 35.1	258.2 ± 372.8	2050.7 ± 2721.2
AFP (ng/mL)					
≤ 7.0	44	73.1 ± 91.7	27.9 ± 38.4	264.2 ± 380.4	2128.1 ± 418.6
> 7.0	10	51.4 ± 55.2	10.4 ± 13.1	128.3 ± 235.3	723.6 ± 1053.4

*SD* standard deviation, *ALT* alanine aminotransferase, *AST* aspartate aminotransferase, *ALB* albumin, *AFP* alpha fetoprotein, *HBV* hepatitis B virus, *HBsAg* hepatitis B surface antigen, *HBeAg* hepatitis B e antigen

^*^p < 0.05, **p < 0.01, compared with data in the next line

### Correlation analyses of breakpoint types, frequencies and enrichments with serum ALT

**Fig. 2:**
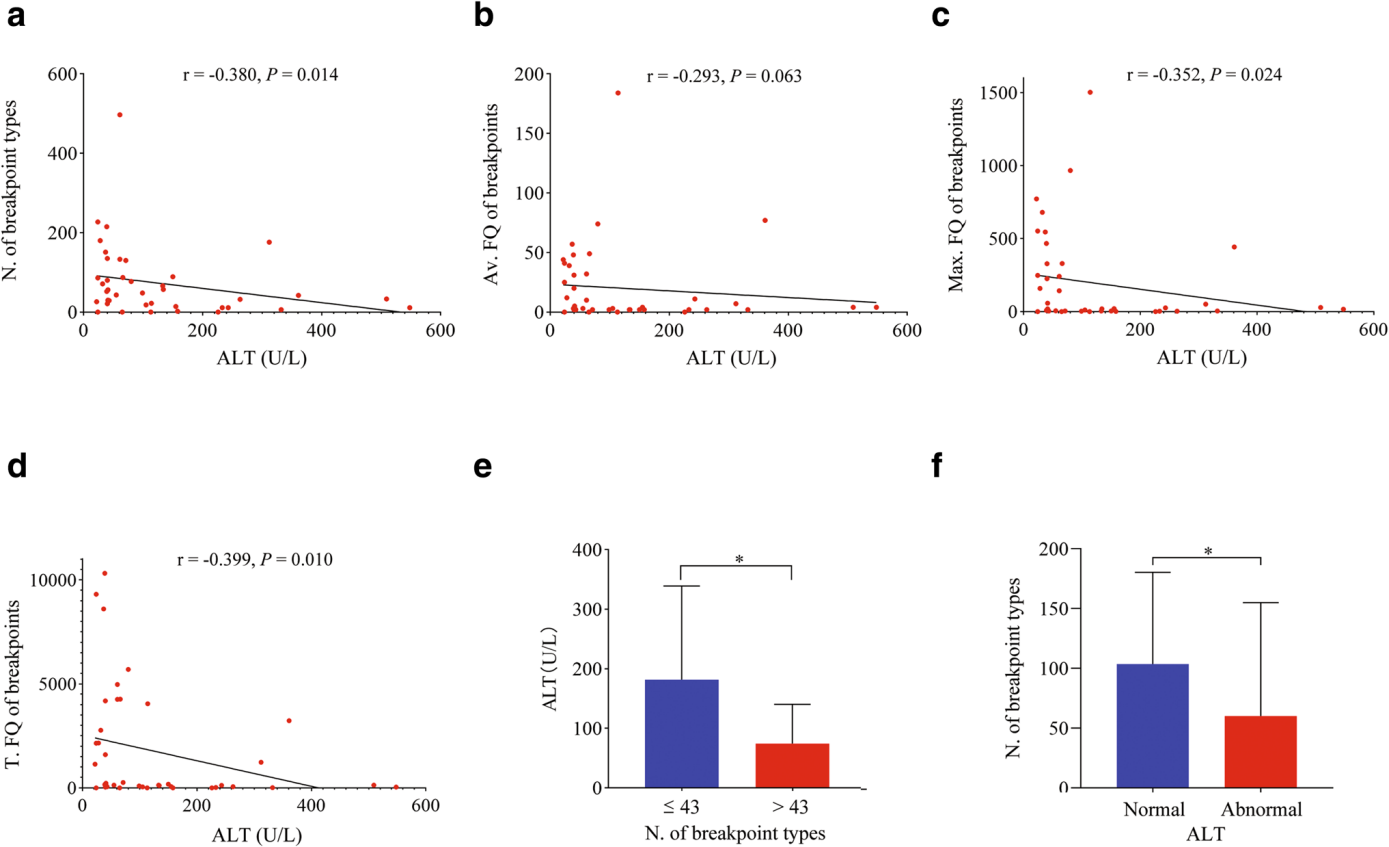
Correlation analyses of breakpoint types and frequencies of HBV integration with ALT in NUC-untreated patients. *N.* number, *FQ* frequency, *Av.* Average, *Max.* maximum, *T.* total; *p < 0 .05. The serum ALT level was negatively correlated with the number of breakpoint types (**a**) and the maximum (**c**) and total (**d**) but not with the average (**b**) frequencies of breakpoints in 41 untreated patients. **e** Patients with fewer breakpoint types had higher levels of serum ALT. **f** Patients with normal ALT had more breakpoint types

ALT and AST were differently associated with the parameters of HBV integration (Table 1). AST had much stronger correlations than ALT (Fig. S3), which may be because some patients received antiviral treatment. Since ALT usually responds to antiviral treatment quickly, 41 NUC-untreated patients were used to further study the correlations of ALT with breakpoint types and frequencies. The integration-related genes were not enriched in the pathway in patients with normal ALT (Fig. S4), but were enriched in gap junction, glutamatergic synapse, and tight junction in patients with abnormal ALT (Fig. S5), suggesting that liver damage increases the chance of HBV integration for some host genes. On the other hand, serum ALT in these patients was found to be negatively correlated with the number of breakpoint types (Fig. 2a) and the maximum (Fig. 2c) and total (Fig. 2d) frequencies of breakpoints but not with the average (Fig. 2b), perhaps being moderated by the change in the number of breakpoint types. Furthermore, patients with fewer breakpoint types had higher serum ALT levels (Fig. 2e), and those with normal ALT levels had more breakpoint types (Fig. 2f). Compared with ALT, AST also had closer correlations with these four parameters in untreated patients (Fig. S6), which was in concordance with the analyses based on all patients (Table 1 and Fig. S3). Therefore, the types, frequencies and enrichments of breakpoints were all influenced by liver damage to some extent. Liver damage mainly eliminates HBV integration and clonal hepatocytes in CHB.

### Correlation analyses of breakpoint types and frequencies with liver histology

**Fig. 3:**
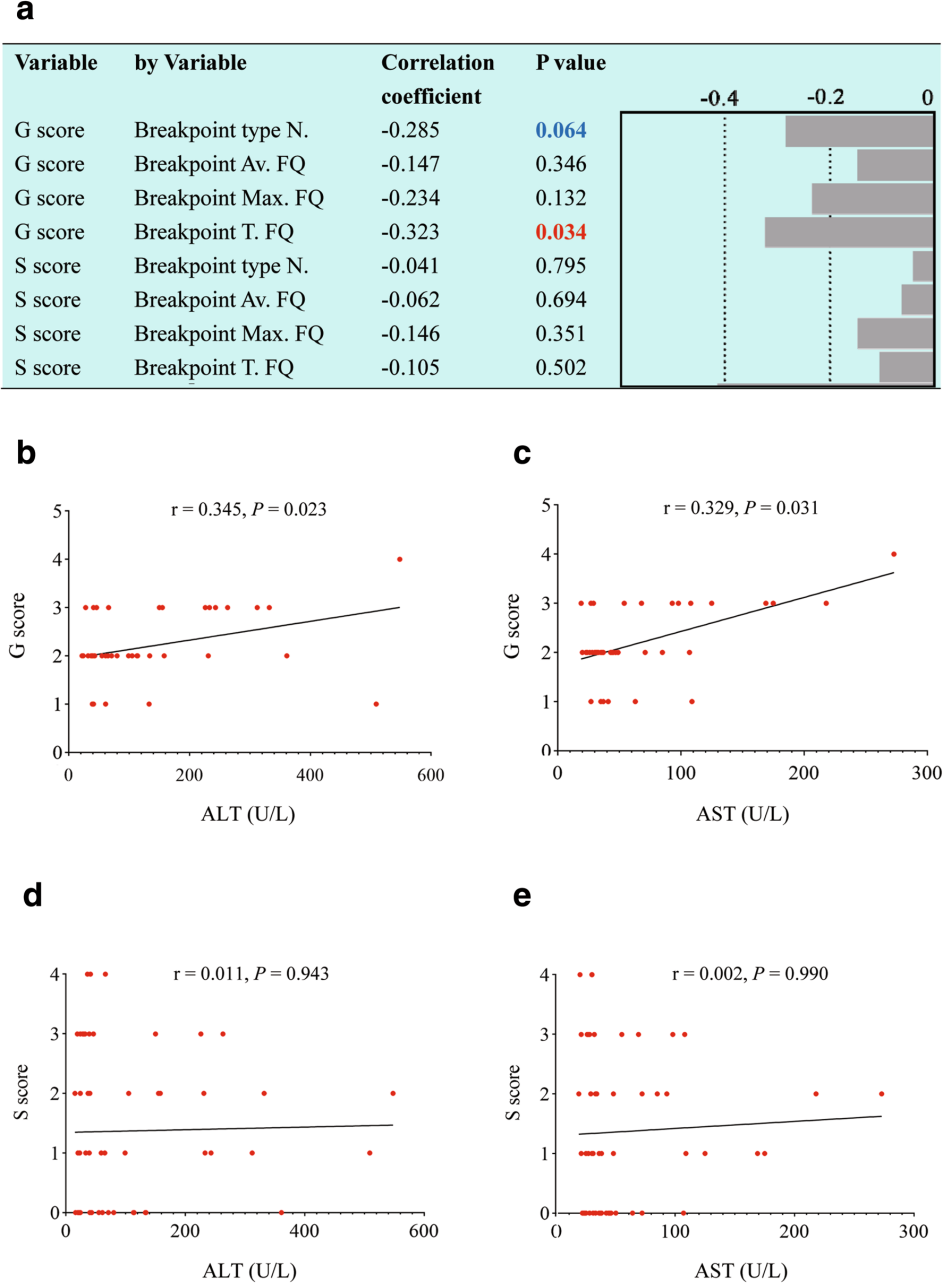
Correlation analyses of breakpoint types and frequencies of HBV integration with liver histology in non-NUC-treated patients. *N.* number, *FQ* frequency, *Av.* Average, *Max.* maximum, *T.* total, *G* necroinflammatory activity grade, *S* fibrosis stage. **a** The G score was marginally correlated with the number of breakpoint types (blue) and significantly negatively correlated with the total frequency (red). The S score was correlated with neither the types nor the frequency of breakpoints. The G (**b** and **c**) score rather than (**d** and **e**) was positively correlated with ALT and AST, respectively

Since the liver histological response to treatment is very slow, 43 non-NUC-treated patients were used to study the correlations of breakpoint types and frequencies with liver histology. The inflammation activity grade (G) scores were marginally correlated with the number of breakpoint types (*p* = 0.064) and significantly negatively correlated with the total frequency, but the fibrosis stage (S) scores were not correlated with any of these four parameters (Fig. 3a). The underlying cause was that the G scores rather than S scores were positively correlated with serum ALT and AST levels (Fig. 3b–e). The correlations of breakpoint types and frequencies with liver histology seemed to be much weaker than those with serum ALT and AST. The G scores were only correlated with the total frequency of breakpoints, perhaps because the total frequency represents the synergistic effect of HBV integration and clonal hepatocyte expansion. Thus, the results of liver histology also support that liver damage mainly eliminates HBV integrations and clonal hepatocytes in CHB. The relationships between the G/S scores and the enrichments of breakpoints in KEGG pathways are shown in Fig. S7 and S8, respectively. Compared with the S scores, the G scores were related with much more pathways, especially in patients with lower G scores (Fig. S7). The S scores, however, were differently related with pathways in patients with low and high S scores (Fig. S8). These results suggest that HBV integration is not only influenced by inflammation crosswise, but also by fibrosis lengthways.

### Correlation analyses of breakpoint types and frequencies with serum HBV DNA load

**Fig. 4:**
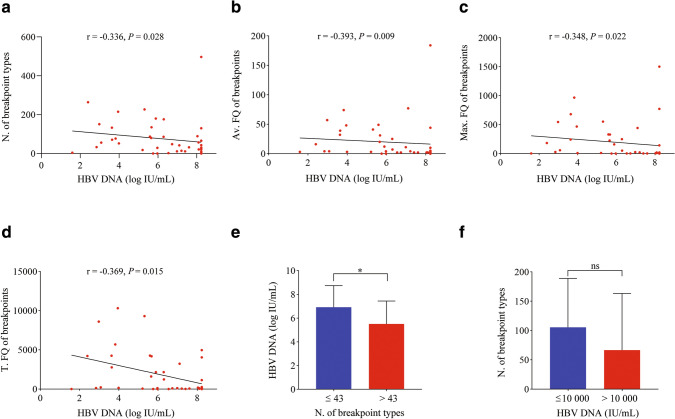
Correlation analyses of the types and frequencies of HBV integration breakpoints with serum HBV DNA load in non-NUC-treated patients. *N.* number, *FQ* frequency, *Av.* average, *Max.* maximum, *T.* total; *p < 0 .05. The serum HBV DNA load was negatively correlated with the number of breakpoint types (**a**) and the average (**b**), maximum (**c**), and total frequencies (**d**) of breakpoints in 41 untreated and 2 temporally treated patients. **e** Patients with more breakpoint types had lower serum HBV DNA load when stratified by the median (43) of breakpoints. **f** There was no difference in the number of breakpoint types when stratified by the HBV DNA load (10,000 IU/mL)

**Fig. 5:**
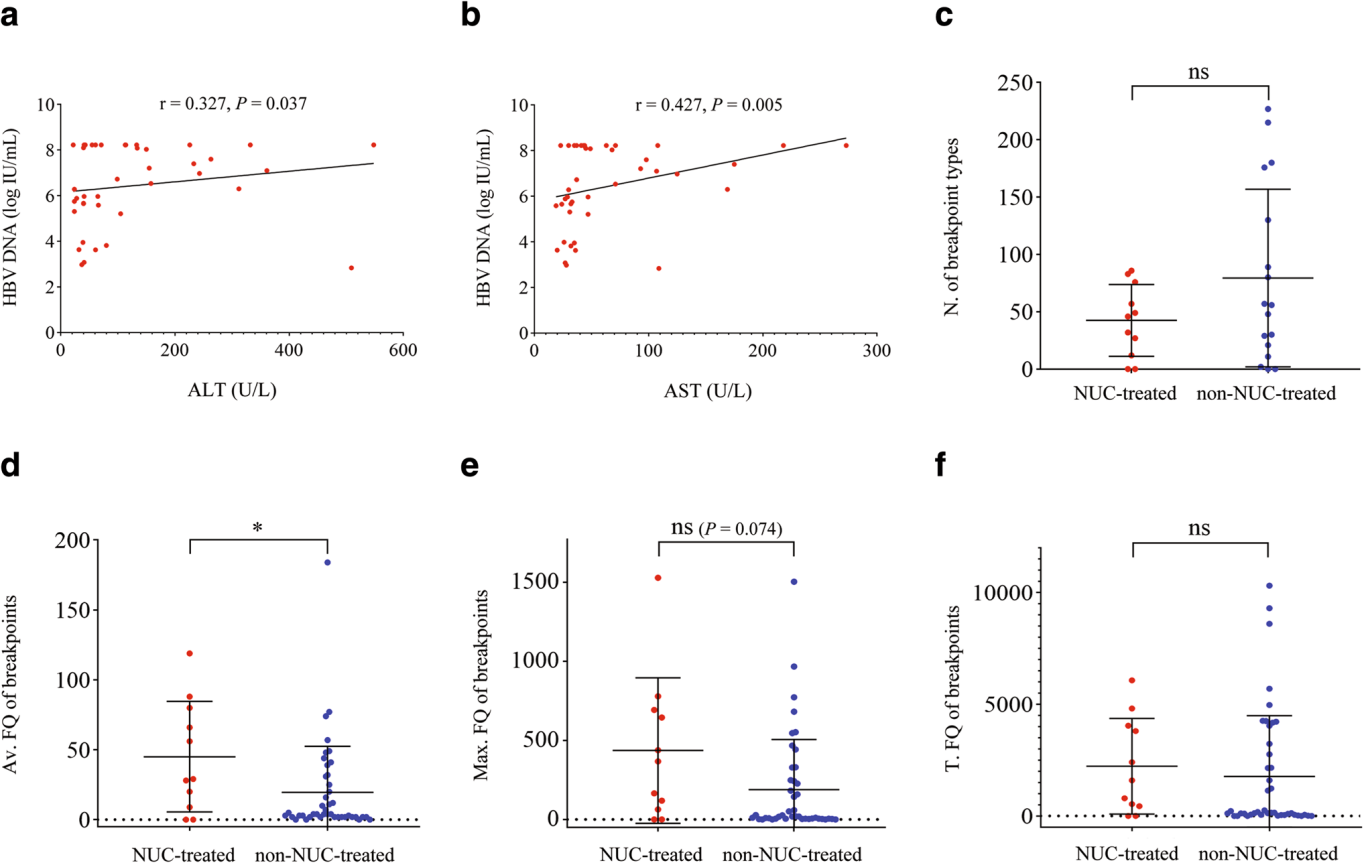
Correlation analyses of HBV DNA with transaminases and influences of NUC treatment on the types, frequencies and enrichments of HBV integration breakpoints. *N.* number, *FQ* frequency, *Av.* average, *Max.* maximum, *T.* total; *p < 0 .05. The serum HBV DNA load was positively correlated with ALT (**a**) and AST (**b**) in NUC-untreated patients. Compared with non-NUC-treated patients, NUC-treated patients had slightly, but statistically insignificantly fewer breakpoint types (**c**), significantly higher average frequency (**d**), marginally higher maximum (**e**) and similar total (**f**) frequencies of breakpoints

HBV DNA load had different correlations with breakpoint types and frequencies (Table 1 and Fig. S3), which may be due to antiviral treatment like ALT. Since HBV DNA load usually responds to the antiviral treatment slowly, 43 non-NUC-treated patients were used to study the above correlations again. The serum HBV DNA load in these patients was negatively correlated with all four parameters of HBV integration (Fig. 4a–d). In addition, patients with more breakpoint types had lower serum viral load when stratified by the median of the breakpoints (Fig. 4e). However, there was no difference in the number of breakpoint types when stratified by the HBV DNA load (Fig. 4f). These results suggest that the number of breakpoint types is also negatively correlated with HBV DNA load, but the negative correlation is not as strong as that of breakpoint frequencies in CHB. Theoretically, the serum HBV DNA load as a direct indicator of HBV replication should be positively correlated with the number of viral integration types. The unexpectedly negative correlation (Table 1, Fig. S3 and Fig. 4) might result from the dominant effect of other important negative impact factors, such as ALT and AST. Indeed, serum HBV DNA load was positively correlated with ALT and AST (Fig. 5a, b), suggesting that the positive influence of HBV DNA load on integration is overwhelmed by the negative influence of liver damage.

### Influence of NUC treatment on breakpoint types, frequencies and enrichments

Compared with non-NUC-treated patients, NUC-treated patients had a statistically nonsignificant decrease in breakpoint types (Fig. 5c) and significantly higher average (Fig. 5d), marginally higher maximum (Fig. 5e, *p* = 0.074) and similar total (Fig. 5f) frequencies of integration breakpoints. The changes in the above parameters in treated patients might be correlated with the significantly lower levels of ALT and AST (Table S3). The high-frequency integration-related genes were significantly different between NUC-treated and non-NUC-treated patients. Moreover, the integration-related genes were not enriched in any pathway in NUC-treated patients (Fig. S9) but were enriched in gap junction, long-term depression, and tight junction in non-NUC-treated patients (Fig. S10). Most of the above results suggest that the remission of liver damage induces the accumulation of clonal hepatocytes, but the dubious decrease in breakpoint types and unchanged total frequency of breakpoints also suggest that the persistent inhibition of HBV replication reduces HBV integration in NUC treatment.

## Discussion

The HBV integration in our CHB patients with hotspots in the region of 1600–1900 bp of the HBV genome and the enrichments to some extent in genes and KEGG pathways are similar to those in most reports [10, 11, 23], suggesting that our cohort of patients is suited for the study of the subsequent fates and evolution of early viral integration in the immune clearance phase. HBV integration mainly shows dispersed distributions in non-tumor tissues [10, 11], and the chromosomal distribution of integrated HBV does not change during HBeAg-seroconversion [23]. However, the HBV integration-related genes in our CHB patients were inclined to gap and tight junctions. These enrichments usually occurred in patients with elevated ALT levels and NUC-untreated patients who usually had significantly higher ALT levels. In contrast, the pathway enrichments may disappear when patients restore to normal ALT levels or receive NUC treatment. These findings suggest that the inventories of HBV integration are dynamically changing with liver damage, and liver damage allows some host genes, especially those genes that are activated for liver repair, to be susceptible to viral integration.

HBV integration events occur in the early stage of HBV infection [12, 13, 24]; as thus, their fates, compared with integration itself, seem to be even more important to the tumorigenesis of HCC. In this study, we found that HBsAg persistence was favorable and serum HBV DNA load, ALT and AST were unfavorable for the maintenance of viral integration. Compared with ALT and the liver S scores, AST and the G scores had closer correlations with the fate of HBV integration, suggesting that necrosis (liver damage) rather than simple hepatocyte degeneration or fibrosis was actually correlated with the fate of HBV integration. The S scores, however, were differently related with pathways in patients with low and high S scores, suggesting that there is a possibility that advanced fibrosis may be correlated with more dangerous HBV integration to HCC hepatocarcinogenesis. The negative correlations with HBV integration in the number of breakpoint types suggest that liver damage is favorable for the elimination of accumulated integrations, and together with the relationships between transaminase levels and KEGG pathways, these results further suggest that liver damage plays a bidirectional role to increase the chance and to reduce the accumulation of HBV integration. However, the major role of liver damage was to reduce the inventory of HBV integration, perhaps due to integration eliminations surpassing chance increases in CHB. Therefore, the number of breakpoint types was weakly, but significantly negatively correlated with liver damage, and with serum HBV DNA load regardless of its enhancing effect on integration chance [25]. Therefore, liver damage is the key determining factor of HBV integration fate in CHB.

The evolution of HBV integration may be another important process of hepatocarcinogenesis. The clone sizes of hepatocytes in patients with HBeAg-negative CHB are much larger than those in patients with HBeAg-positive CHB [23]. When breakpoint frequencies were used as indicators of clonal expansion in this study, a similar phenomenon evidenced by elevated average and maximum frequencies of breakpoints was found in our HBeAg-negative CHB patients. Moreover, some hepatocytes were found to clonally expand to more than 10^3^ in scale. The average, maximum and total breakpoint frequencies were all negatively correlated with the liver damage surrogate indicators of ALT, AST and liver inflammation activity grade score, suggesting that liver damage eliminates clonal hepatocytes in CHB. However, many scientists believe that the immune killing of infected hepatocytes is the strongest known pressure point to drive clonal hepatocyte expansion [14], suggesting that liver damage may also bidirectionally influence clonal hepatocyte expansion but may primarily help to eliminate clonal hepatocytes in CHB.

The underlying mechanism of HCC prevalence despite sustained viral response and remission of liver inflammation during NUC therapy has been a hot topic in recent years [1–5]. In this study, compared with non-NUC-treated patients, treated patients only had a minor decrease in breakpoint types but had significantly higher average and marginally higher maximum frequencies of breakpoints, implying an increase in clonal hepatocytes, though the total frequency was similar because it was minimized by the small decrease in breakpoint types. The remission of liver damage explains the increase in clonal hepatocytes but cannot explain the decrease in breakpoint types since it should increase because of elimination reduction. Therefore, the decrease in breakpoint types may result from persistent HBV replication inhibition that leads to a reduction in the chance of integration and a subsequent decrease in the inventory of viral integration. Together with the significant increase in clonal hepatocytes, the decrease in breakpoint types may explain why NUC treatment reduces but does not rule out the risk of HCC, especially in the later stage when clonal hepatocyte expansion is almost finished, supporting the views of some scientists to conduct antiviral intervention early to reduce genetic damage to a large extent [12, 14].

Integrated HBV DNA has the potential to express HBsAg, which is unfavorable for a radical cure [26, 27]. Therefore, knowing how to remove HBV integrations is favorable not only for HCC prevention but also for the radical cure of HBV infection. The significance of liver damage to HBV integration and clonal hepatocyte expansion in this study supports the restoration of host antiviral immunity [28]. Excitingly, neoadjuvant anti-PD1 therapy significantly decreases HBsAg, especially when there is a flare of ALT, in patients with virally suppressed CHB [29]. However, persistent inflammation in the liver promotes clonal hepatocyte expansion and increases the risk of HCC [14, 30]. Therefore, much work still needs to be done before we are able to use host antiviral immunity to prevent HCC. Nonetheless, transient and limited inflammation may benefit CHB patients in either HCC prevention or radical cure of HBV infection.

In conclusion, our study uncovered the characteristics of HBV integration in CHB patients and provided convincing evidence that liver damage increased the chance of HBV integration but might mainly prevent HCC by removing viral integrations and clonal hepatocytes in CHB. NUC treatment may reduce the chance of HBV integration but simultaneously reduce the elimination of viral integration and clonal hepatocytes. These results might have many implications for CHB treatments and HCC prevention in the future.

## Supplementary Information

Below is the link to the electronic supplementary material.

Supplementary file1 (DOCX 27016 KB)

## Data Availability

All data generated or used during the study are available from the corresponding author by request.

## References

[1] Choi J, Han S, Kim N, Lim YS (2017). Increasing burden of liver cancer despite extensive use of antiviral agents in a hepatitis B virus-endemic population. Hepatology.

[2] Papatheodoridis GV, Idilman R, Dalekos GN, Buti M, Chi H, Boemmel FV (2017). The risk of hepatocellular carcinoma decreases after the first 5 years of entecavir or tenofovir in Caucasians with CHB. Hepatology.

[3] Kim SU, Seo YS, Lee HA, Kim MN, Lee EJ, Shin HJ (2020). Hepatocellular carcinoma risk steadily persists over time despite long-term antiviral therapy for hepatitis B: a multicenter study. Cancer Epidemiol Biomarkers Prev.

[4] Grossi G, Viganò M, Loglio A, Lampertico P (2017). Hepatitis B virus long-term impact of antiviral therapy nucleot(s)ide analogues (NUCs). Liver Int.

[5] Arends P, Sonneveld MJ, Zoutendijk R, Carey I, Brown A, Fasano M (2015). Entecavir treatment does not eliminate the risk of hepatocellular carcinoma in chronic hepatitis B: limited role for risk scores in Caucasians. Gut.

[6] Sinn DH, Kim SE, Kim BK, Kim JH, Choi MS (2019). The risk of hepatocellular carcinoma among chronic hepatitis B virus-infected patients outside current treatment criteria. J Viral Hepat.

[7] Yip TC, Chan HL, Wong VW, Tse YK, Lam KL, Wong GL (2017). Impact of age and gender on risk of hepatocellular carcinoma after hepatitis B surface antigen seroclearance. J Hepatol.

[8] Chen Y, Tian Z (2019). HBV-Induced Immune Imbalance in the Development of HCC. Front Immunol.

[9] Budzinska MA, Shackel NA, Urban S, Tu T (2018). Cellular genomic sites of hepatitis B virus DNA integration. Genes (Basel).

[10] Zhao LH, Liu X, Yan HX, Li WY, Zeng X, Yang Y (2016). Genomic and oncogenic preference of HBV integration in hepatocellular carcinoma. Nat Commun.

[11] Sung WK, Zheng H, Li S, Chen R, Liu X, Li Y (2012). Genome-wide survey of recurrent HBV integration in hepatocellular carcinoma. Nat Genet.

[12] Mason WS, Gill US, Litwin S, Zhou Y, Peri S, Pop O (2016). HBV DNA integration and clonal hepatocyte expansion in chronic hepatitis b patients considered immune tolerant. Gastroenterology.

[13] Chauhan R, Churchill ND, Mulrooney-Cousins PM, Michalak TI (2017). Initial sites of hepadnavirus integration into host genome in human hepatocytes and in the woodchuck model of hepatitis B-associated hepatocellular carcinoma. Oncogenesis.

[14] Zoulim F, Mason WS (2012). Reasons to consider earlier treatment of chronic HBV infections. Gut.

[15] Mason WS, Liu C, Aldrich CE, Litwin S, Yeh MM (2010). Clonal expansion of normal-appearing human hepatocytes during chronic hepatitis B virus infection. J Virol.

[16] Brunner SF, Roberts ND, Wylie LA, Moore L, Aitken SJ, Davies SE (2019). Somatic mutations and clonal dynamics in healthy and cirrhotic human liver. Nature.

[17] Tu T, Mason WS, Clouston AD, Shackel NA, McCaughan GW, Yeh MM (2015). Clonal expansion of hepatocytes with a selective advantage occurs during all stages of chronic hepatitis B virus infection. J Viral Hepat.

[18] Li W, Zeng X, Lee NP, Liu X, Chen S, Guo B (2013). HIVID: an efficient method to detect HBV integration using low coverage sequencing. Genomics.

[19] Wang K, Li M, Hakonarson H (2010). ANNOVAR: functional annotation of genetic variants from high-throughput sequencing data. Nucleic Acids Res.

[20] Li L, Zhang JA, Dose M, Kueh HY, Mosadeghi R, Gounari F (2013). A far downstream enhancer for murine Bcl11b controls its T-cell specific expression. Blood.

[21] Hu Z, Zhu D, Wang W, Li W, Jia W, Zeng X (2015). Genome-wide profiling of HPV integration in cervical cancer identifies clustered genomic hot spots and a potential microhomology-mediated integration mechanism. Nat Genet.

[22] Yu G, Wang LG, Han Y, He QY (2012). clusterProfiler: an R package for comparing biological themes among gene clusters. OMICS.

[23] Budzinska MA, Shackel NA, Urban S, Tu T (2018). Sequence analysis of integrated hepatitis B virus DNA during HBeAg-seroconversion. Emerg Microbes Infect.

[24] Tu T, Budzinska MA, Vondran FWR, Shackel NA, Urban S (2018). Hepatitis B virus DNA integration occurs early in the viral life cycle in an in vitro infection model via sodium taurocholate cotransporting polypeptide-dependent uptake of enveloped virus particles. J Virol.

[25] Ruan P, Dai X, Sun J, He C, Huang C, Zhou R (2019). Different types of viral-host junction found in HBV integration breakpoints in HBV-infected patients. Mol Med Rep.

[26] Tu T, Budzinska MA, Shackel NA, Urban S (2017). HBV DNA integration: molecular mechanisms and clinical implications. Viruses.

[27] Hu B, Wang R, Fu J, Su M, Du M, Liu Y (2018). Integration of hepatitis B virus S gene impacts on hepatitis B surface antigen levels in patients with antiviral therapy. J Gastroenterol Hepatol.

[28] Durantel D, Zoulim F (2016). New antiviral targets for innovative treatment concepts for hepatitis B virus and hepatitis delta virus. J Hepatol.

[29] Gane E, Verdon DJ, Brooks AE, Gaggar A, Nguyen AH, Subramanian GM (2019). Anti-PD-1 blockade with nivolumab with and without therapeutic vaccination for virally suppressed chronic hepatitis B: a pilot study. J Hepatol.

[30] Peters MG, Hepatitis B (2019). Virus infection: what is current and new. Top Antivir Med.

