# Supplementary material for: Liver damage favors the eliminations of HBV integration and clonal hepatocytes in chronic hepatitis B
Source: Hepatol Int. 2021 Feb 3;15(1):60–70. doi: 10.1007/s12072-020-10125-y (PMC7886763; doi:10.1007/s12072-020-10125-y)
Supplement: Supplementary file 1 — Supplementary file1 (DOCX 27016 KB) [file 12072_2020_10125_MOESM1_ESM.docx]

**Supplementary Materials**

**Table S1. Demographic and clinical characteristics of 54 chronic hepatitis B patients**

| **Variables** | **Mean ± SD / n (%)** | **Variables** | **Mean ± SD / n (%)** |
| --- | --- | --- | --- |
| Age (years) | 36.2±8.8 | HBV genotypes |  |
| Gender |  | B | 16 (29.6) |
| Male | 39 (72.2) | B/C | 28 (51.9) |
| Female | 15 (27.8) | C | 10 (18.5) |
| ALT (U/L) | 107.2±121.4 | HBsAg (+) (years) |  |
| AST (U/L) | 56.6±52.2 | ≤15 | 31 (57.4) |
| γGT (U/L) | 46.0±52.5 | >15 | 23 (42.6) |
| TBil (μmoL/L) | 15.7±7.9 | HBsAg (IU/mL) |  |
| ALB (g/L) | 43.95±3.18 | <100 | 1 ( 1.9) |
| PT (s) | 11.6±0.9 | 100-1 000 | 10 (18.5) |
| AFP (ng/mL) | 9.5±24.8 | >1 000 | 43 (79.6) |
| Serum Cr (μmol/L) | 68.0±12.9 | HBeAg |  |
| PLT (×10⁹/L) | 209.2±65.4 | (+) | 33 (61.1) |
| Liver biopsy |  | (-) | 21 (38.9) |
| G | 2.13±0.65 | HBV DNA (copies) |  |
| S | 1.37±1.29 | ≤10 000 | 21 38.9) |
| CHB severity ^a^ |  | >10 000 | 33 (61.1) |
| Mild | 35 (64.8) | Antivirus therapy ^b^ |  |
| Moderate | 15 (27.8) | No | 43 (79.6) |
| Severe | 4 (7.4) | Yes | 11 (20.4) |

^a^ Mild (G ≤ 2 and S ≤ 1), moderate (2 < G ≤ 3 or S = 2) and severe types (G = 4 or S ≥ 3) based on the pathological findings; ^b^ Received antiviral therapy for more than 3 months. SD, standard deviation; ALT, alanine aminotransferase; AST, aspartate aminotransferase; γGT, γ glutamyl transpeptidase; TBil, total bilirubin; ALB, albumin; PT, prothrombin time; AFP, alpha fetoprotein; CR, creatinine; PLT, platelet; G, inflammation activity grade; S, fibrosis stage; HBV, hepatitis B virus; HBsAg, hepatitis B surface antigen; HBeAg, hepatitis B e antigen.

**Table S2. General information about antiviral treatment**

| **Categories** | **Patients** | **Antivirals** | **Duration**  (months) |
| --- | --- | --- | --- |
| Temporally-treated |  |  |  |
|  | 1 | Entecavir | 1 |
|  | 2 | Entecavir | 3 |
| Treated |  |  |  |
|  | 1 | Entecavir | 4 |
|  | 2 | Entecavir | 10 |
|  | 3 | Entecavir | 12 |
|  | 4 | Entecavir | 18 |
|  |  | Switch to tenofovir disoproxil | 6 |
|  | 5 | Adefovir | 24 |
|  | 6 | Entecavir | 24 |
|  | 7 | Entecavir | 24 |
|  | 8 | Entecavir | 36 |
|  |  | Switch to tenofovir disoproxil | 5 |
|  | 9 | Adefovir | 60 |
|  | 10 | Lamivudine | 20 |
|  |  | Switch to adefovir | 22 |
|  |  | Switch to tenofovir disoproxil | 18 |
|  | 11 | Lamivudine | 72 |
|  |  | Add on adefovir | 24 |

**Table S3. Comparisons of demographic and clinical characteristics in patients with or without antiviral treatment**

| **Variables** | **NUC-treated**  **Mean ± SD / n (%)** | **Non-NUC-treated**  **Mean ± SD / n (%)** | ***P* value** |
| --- | --- | --- | --- |
| Age (years) | 42.00±11.60 | 34.74±7.42 | 0.072 |
| Gender |  |  | 0.241 |
| Male | 10 (25.64) | 29 (74.36) |  |
| Female | 1 (6.67) | 14 (93.33) |  |
| ALT (U/L) | 28.09±13.04 | 127.42±128.41 | <0.001 |
| AST (U/L) | 24.00±4.15 | 64.98±55.57 | <0.001 |
| γGT (U/L) | 32.00±19.82 | 49.58±57.64 | 0.420 |
| TBil (μmoL/L) | 13.34±6.08 | 16.24±8.27 | 0.186 |
| ALB (g/L) | 43.45±2.76 | 44.09±3.30 | 0.553 |
| PT (s) | 11.43±0.73 | 11.67±0.89 | 0.416 |
| AFP (ng/mL) | 4.17±2.60 | 10.83±27.65 | 0.83 |
| Serum Cr (μmol/L) | 72.82±8.08 | 66.79±13.63 | 0.168 |
| PLT (×10⁹/L) | 193.91±65.82 | 213.14±65.53 | 0.384 |
| Liver biopsy |  |  |  |
| G | 1.91±0.30 | 2.19±0.70 | 0.181 |
| S | 1.55±1.29 | 1.33±1.30 | 0.541 |
| CHB severity |  |  | 0.807 |
| Mild | 8 (22.86) | 27 (77.14) |  |
| Moderate | 2 (13.33) | 13 (86.67) |  |
| Severe | 1 (25.00) | 3 (75.00) |  |

NUC, nucleos(t)ide analogues; NUC-treated, received NUC therapy for more than 3 months; non-NUC-treated, untreated or received NUC therapy for less than 3 months. ALT, alanine aminotransferase; AST, aspartate aminotransferase; γGT, γ glutamyl transpeptidase; TBil, total bilirubin; ALB, albumin; PT, prothrombin time; AFP, alpha fetoprotein; CR, creatinine; PLT, platelet; G, inflammation activity grade; S, fibrosis stage.

**
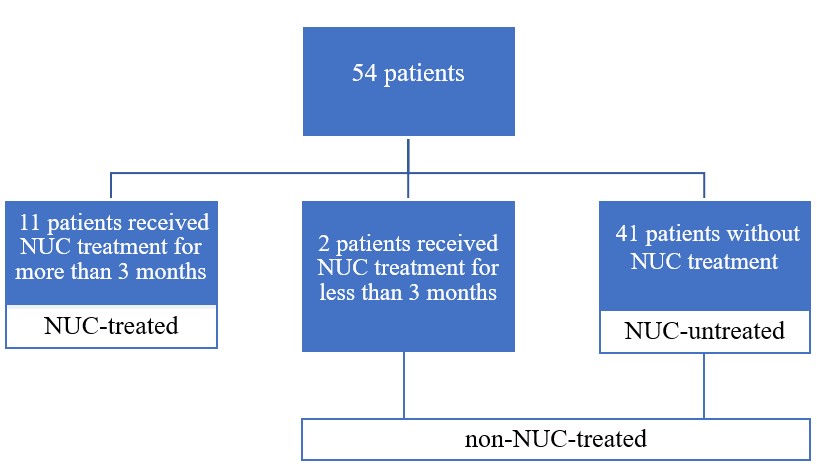
**

**Figure S1. Definition of patient groups.**


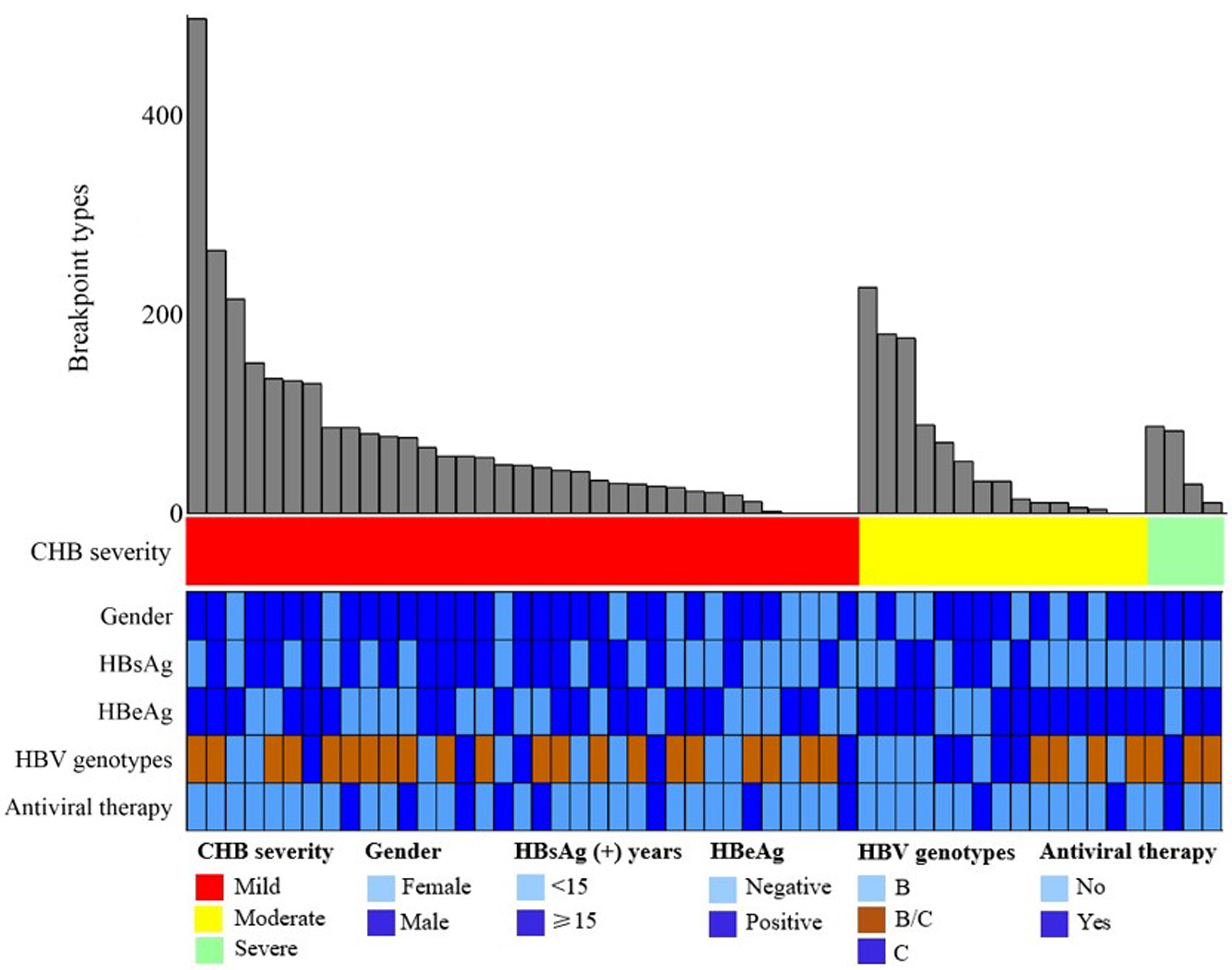


**Figure S2. Clinical annotation of HBV integration breakpoints in human genome in 54 CHB patients.** All panels are aligned with vertical tracks representing 54 individuals. CHB, chronic hepatitis B; HBsAg, hepatitis B surface antigen; HBeAg, hepatitis B e antigen; HBV, hepatitis B virus; Antiviral therapy, treated with nucleos(t)ide analogues.


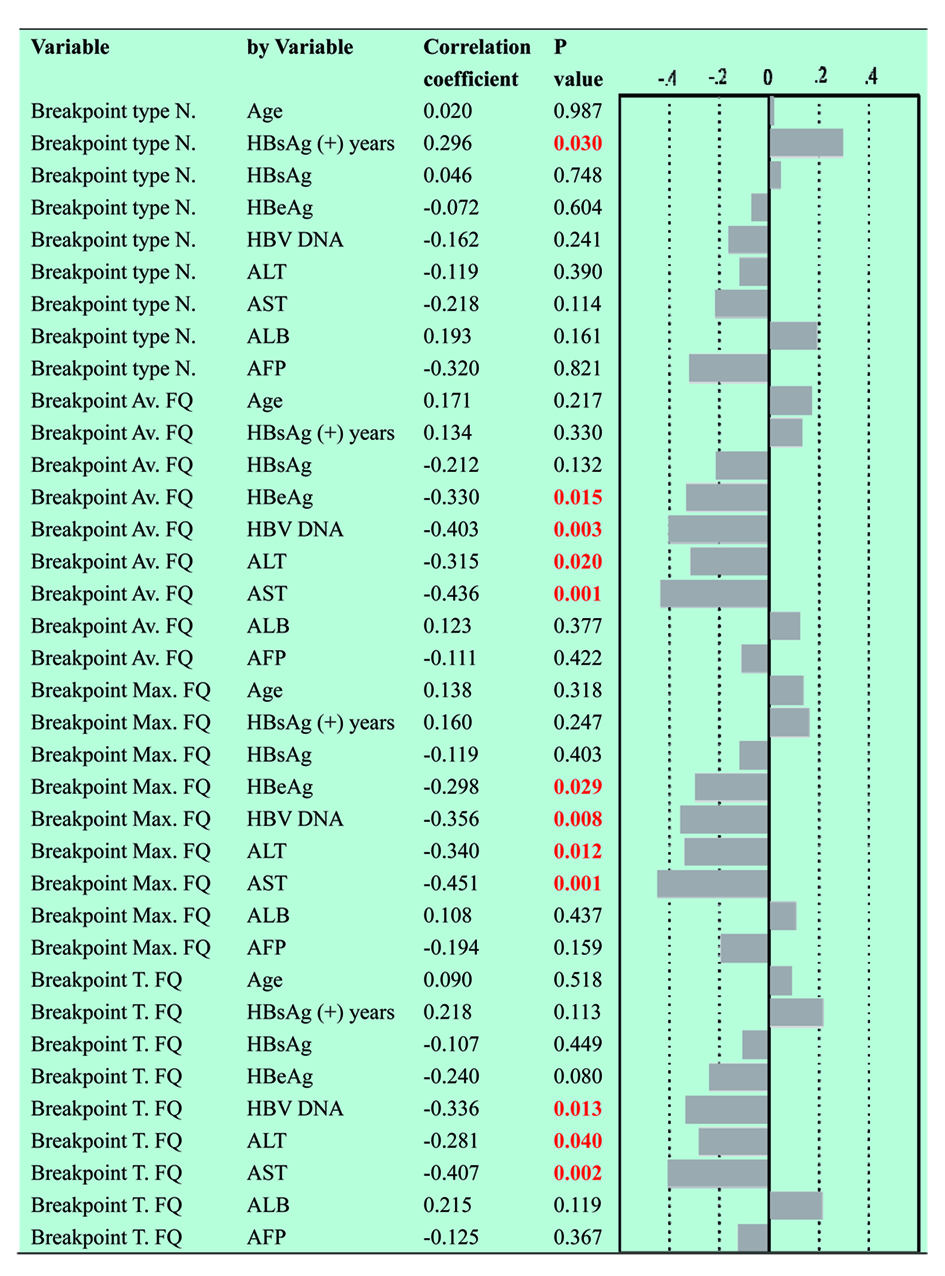


**Figure S3. Correlation analyses of the types and frequencies of HBV integration breakpoints with clinical data in 54 CHB patients.** N., number; FQ, frequency; Av., average; Max., maximum; T., total. HBsAg, hepatitis B surface antigen; HBeAg, hepatitis B e antigen; HBV, hepatitis B virus; ALT, alanine aminotransferase; AST, aspartate aminotransferase; ALB, albumin; AFP, alpha fetoprotein. Red characters indicate statistical significances.


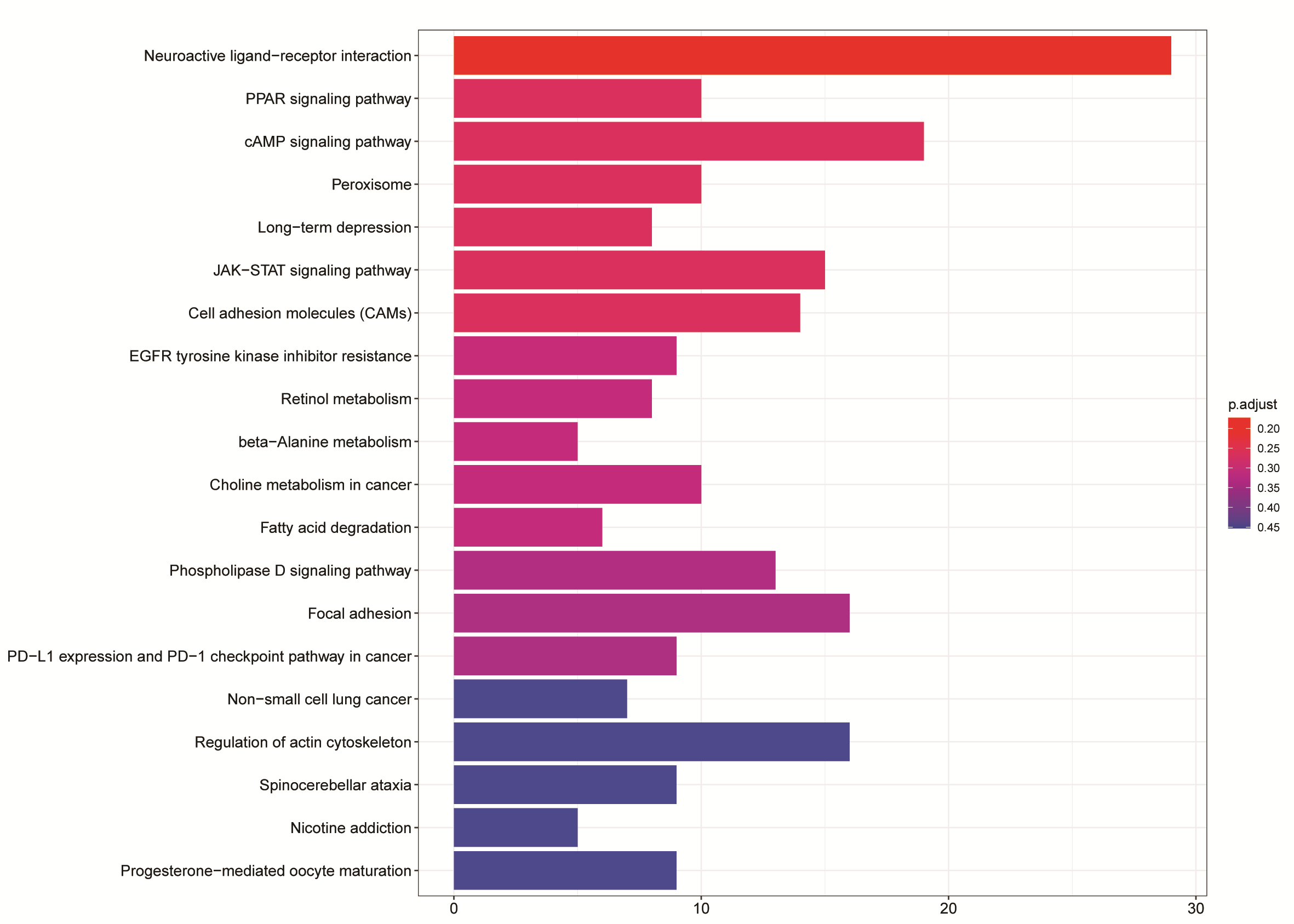

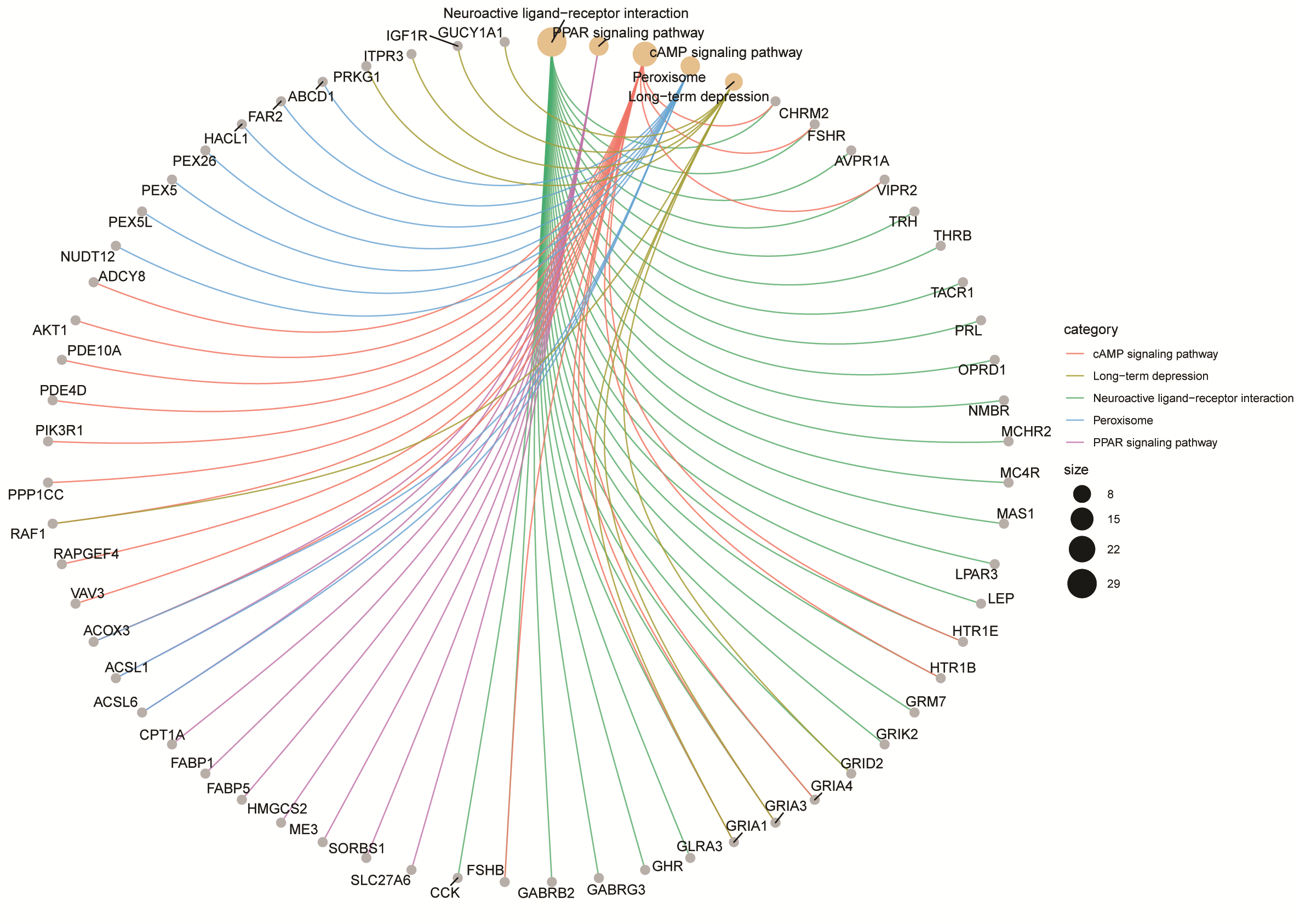


**Figure S4. No enrichments of HBV integration breakpoints in KEGG pathways in NUC-untreated patients with normal ALT.** (A) Histogram of HBV integration breakpoints in KEGG pathways. The vertical and horizontal axes illustrate the name of pathway and the count of the enriched genes, respectively. The color shows the statistical significance with red relating to a smaller *P*-value. (B) Network map of the pathways and their HBV integration-related genes. Gray node in the figure represents a gene; Orange node represents the pathway and the circle size represented gene numbers in the pathway; Color line between the nodes represented the connection of pathways and genes.


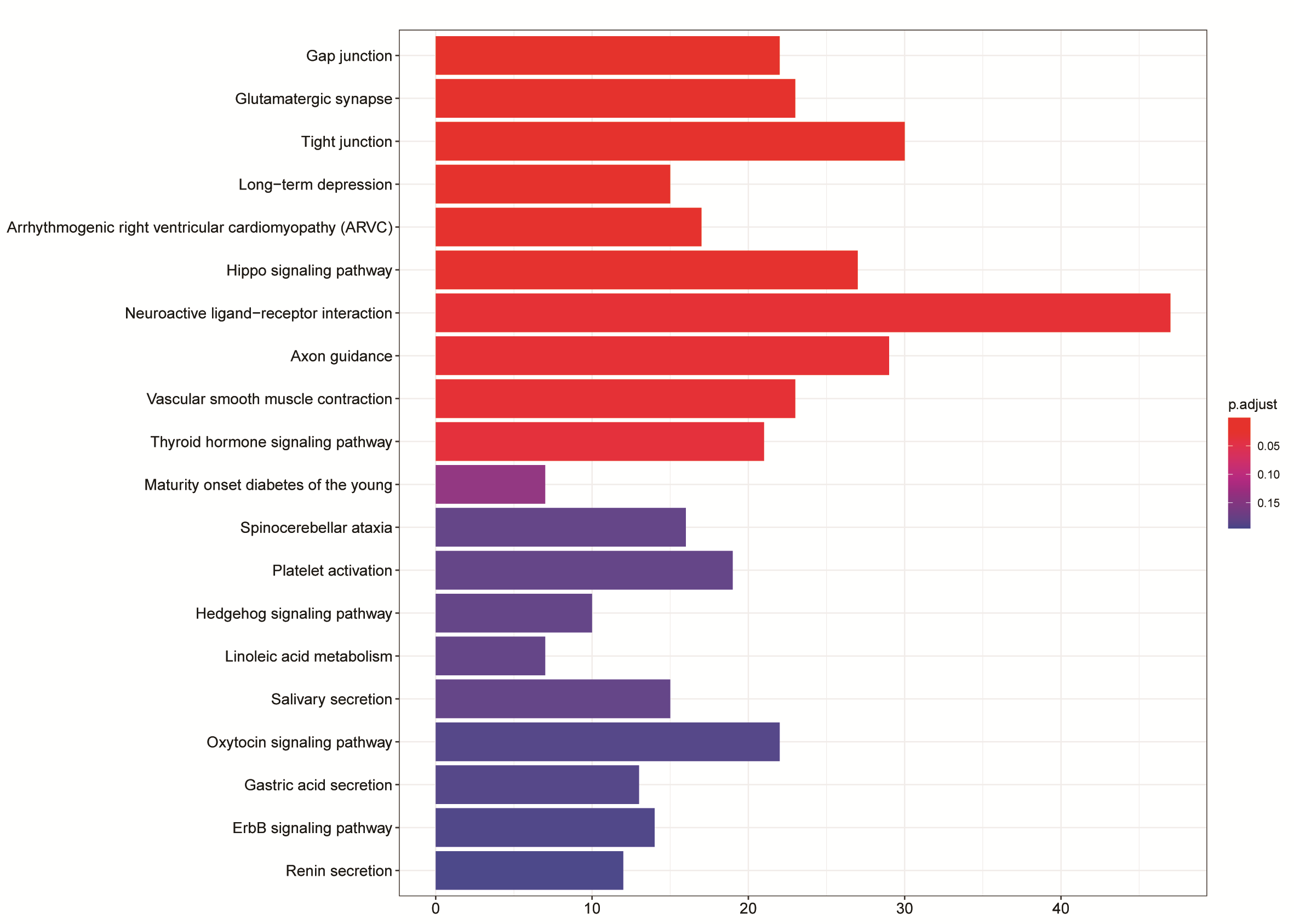

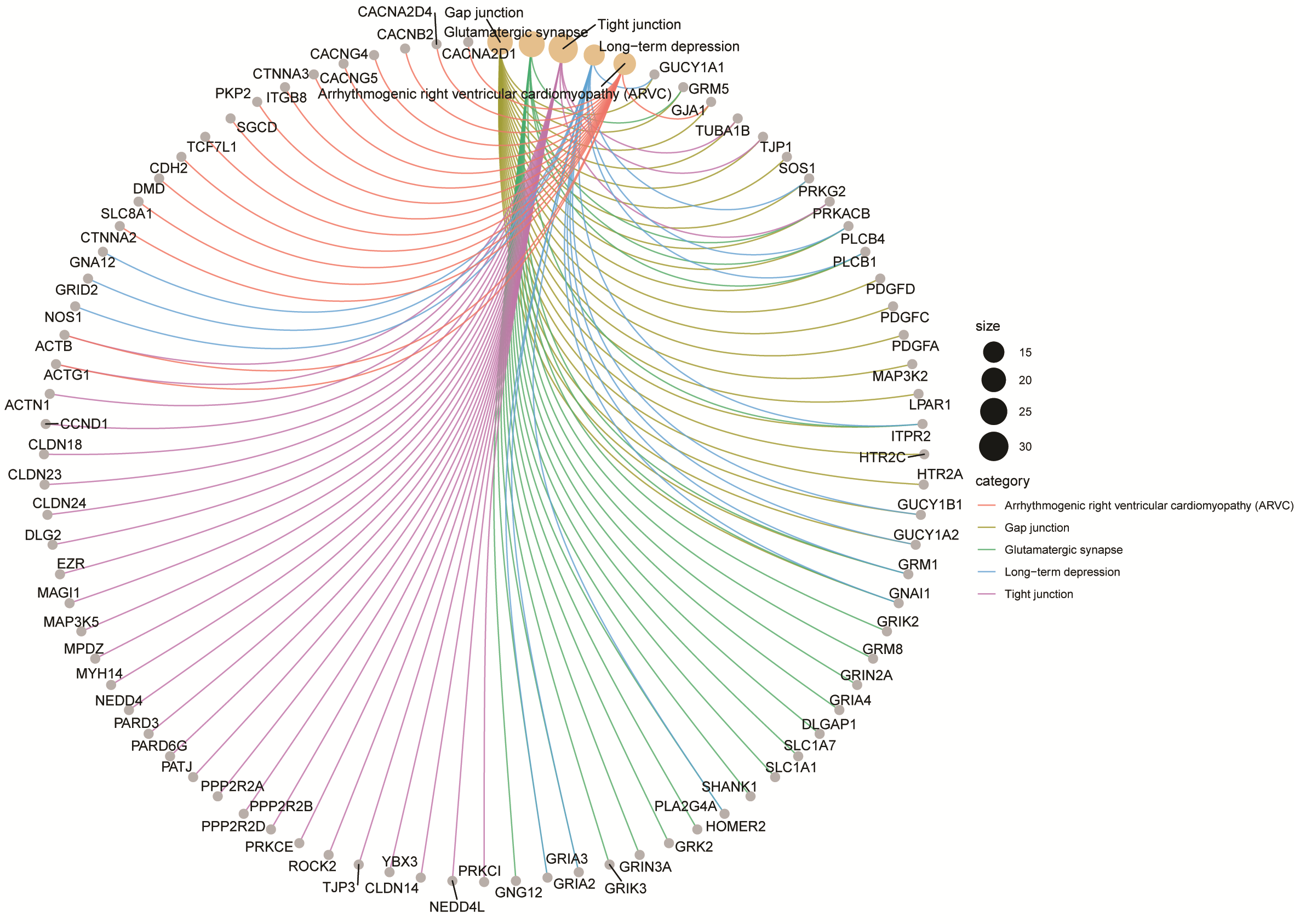


**Figure S5. Enrichments of HBV integration breakpoints in KEGG pathways in NUC-untreated patients with abnormal ALT.** (A) Histogram of HBV integration breakpoints in KEGG pathways. The vertical and horizontal axes illustrate the name of pathway and the count of the enriched genes, respectively. The color shows the statistical significance with red relating to a smaller *P*-value. (B) Network map of the pathways and their HBV integration-related genes. Gray node in the figure represents a gene; Orange node represents the pathway and the circle size represented gene numbers in the pathway; Color line between the nodes represented the connection of pathways and genes.


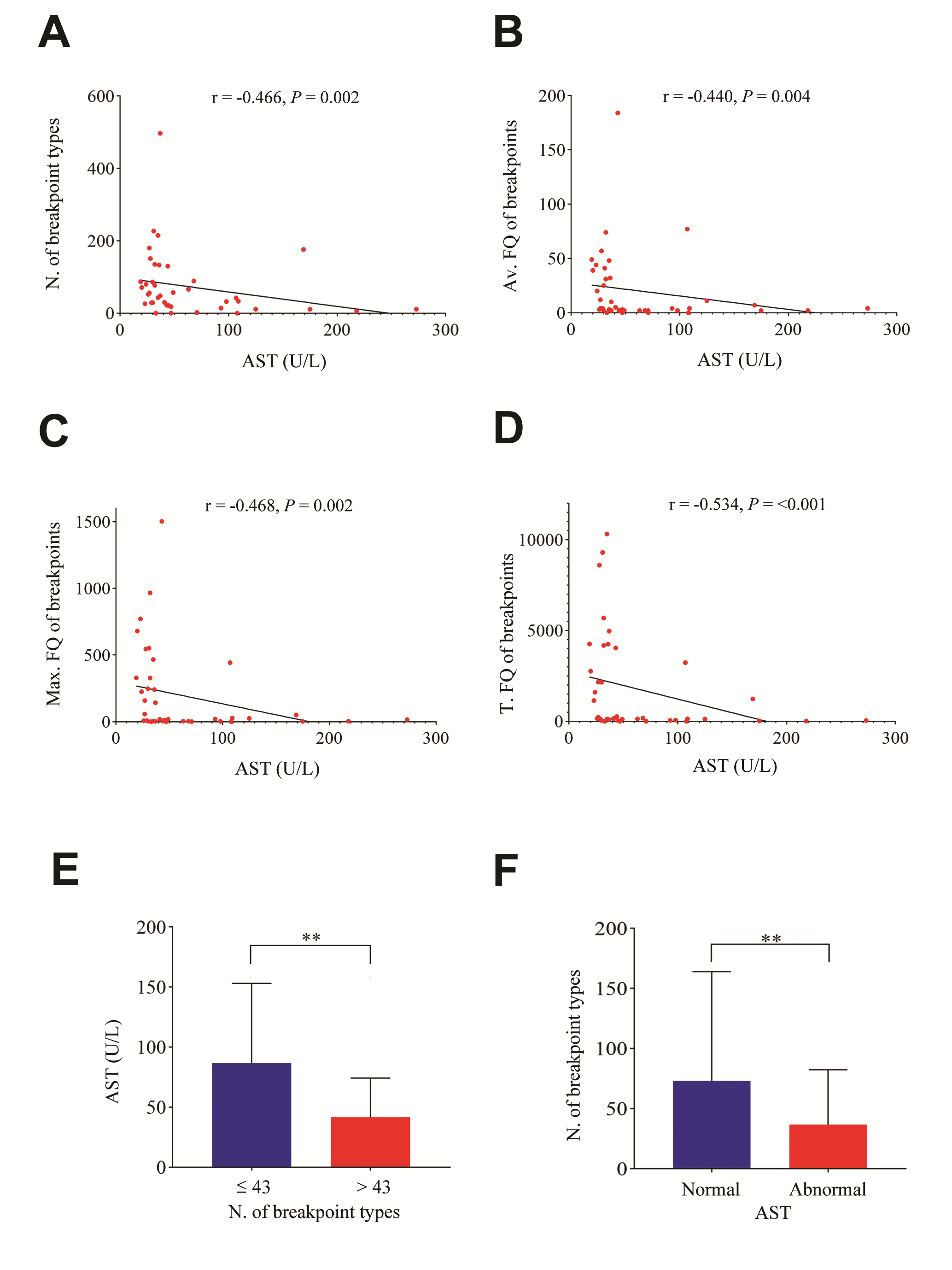


**Figure S6. Correlation analyses of breakpoint types and frequencies of HBV integration with AST in NUC-untreated patients.** N., number; FQ, frequency; Av., average; Max., maximum; T., total; * *P* <0 .05, ** *P* <0 .01. Each red point represents one patient. The serum AST level was negatively correlated with the number of breakpoint types (A) and the average (B), maximum (C), and total frequencies (D) of breakpoints in 41 NUC-untreated patients. (E) Patients with less breakpoint types had higher levels of serum AST. (F) Patients with normal AST had more breakpoint types.


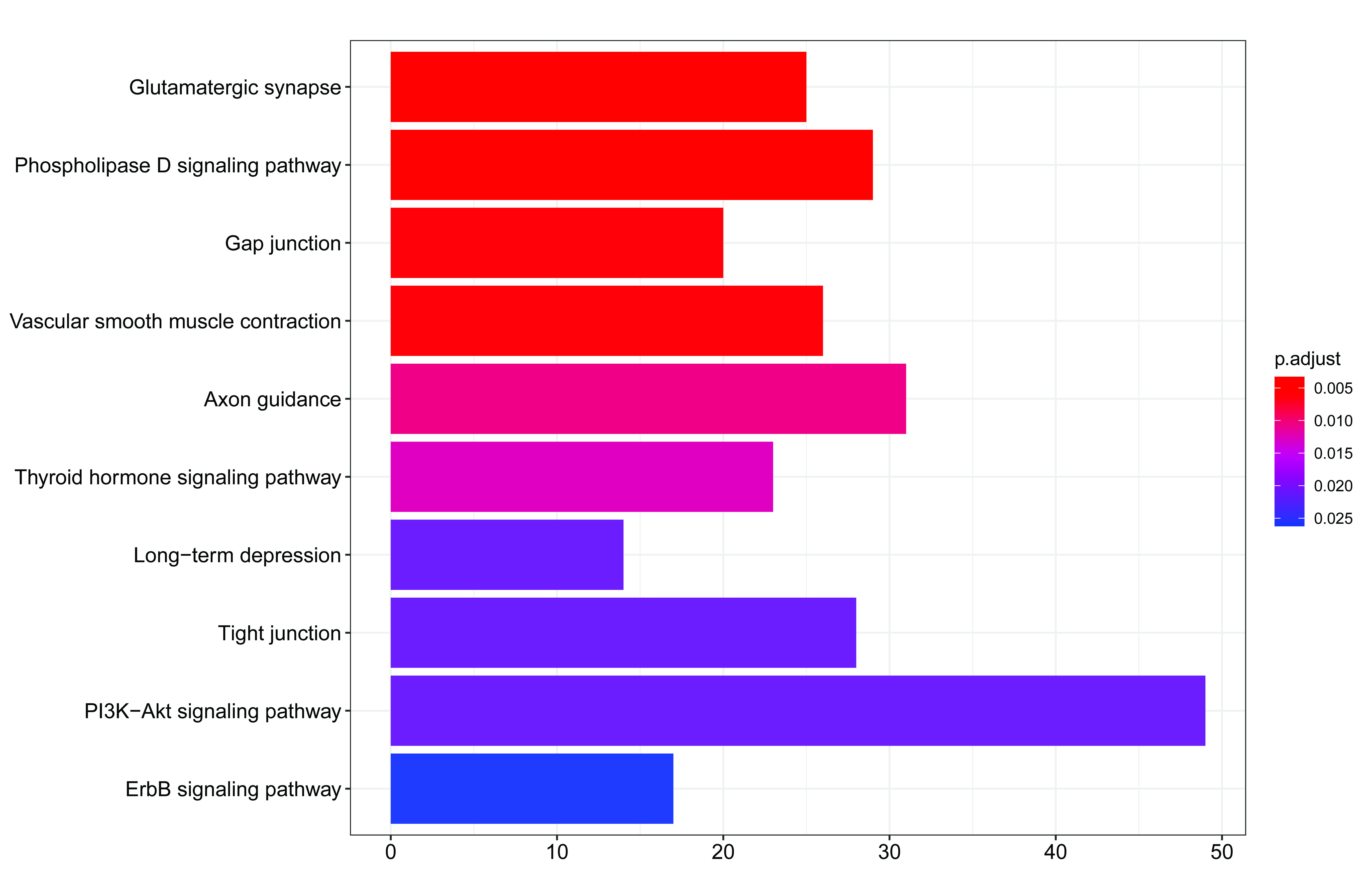

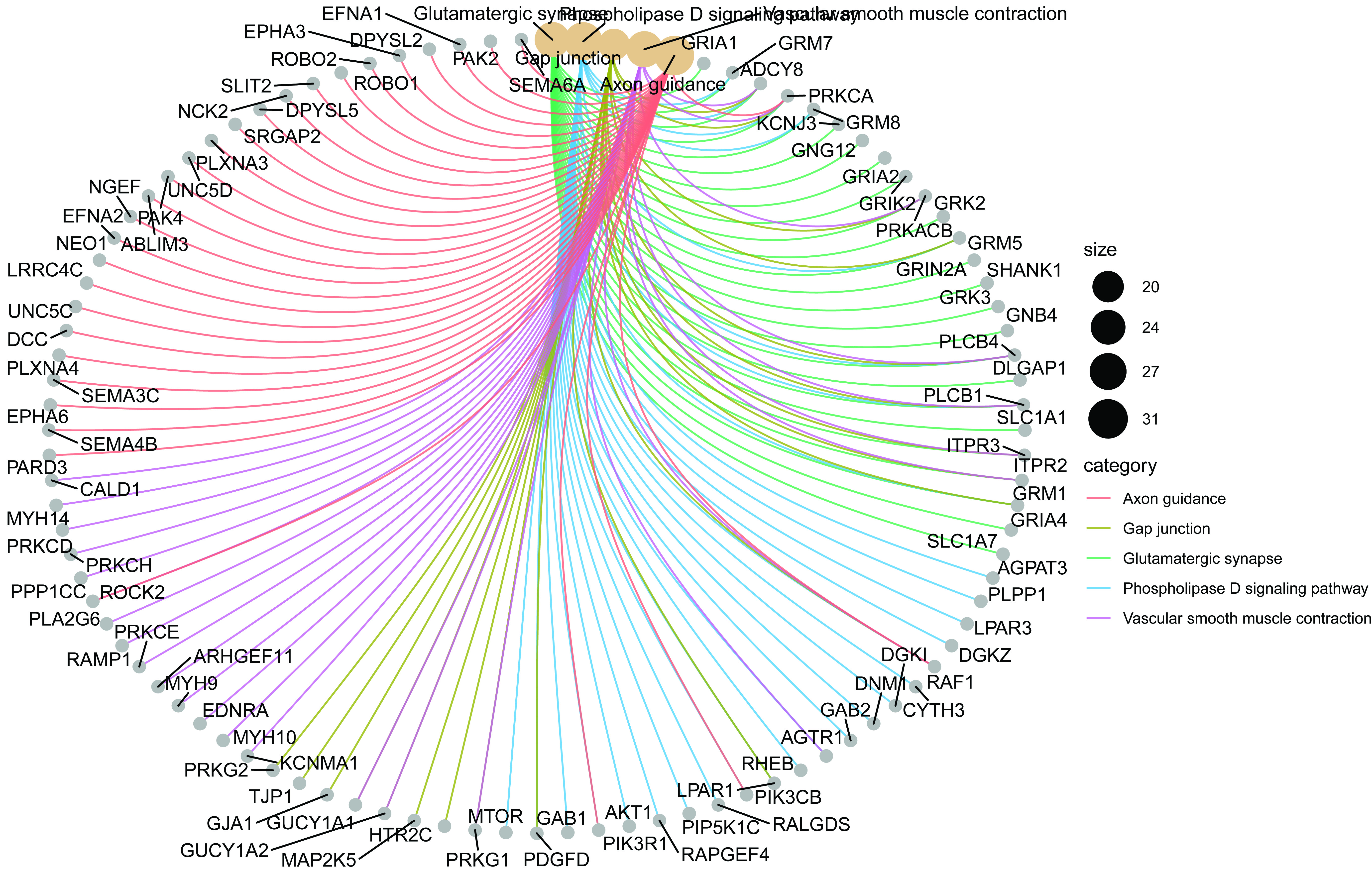

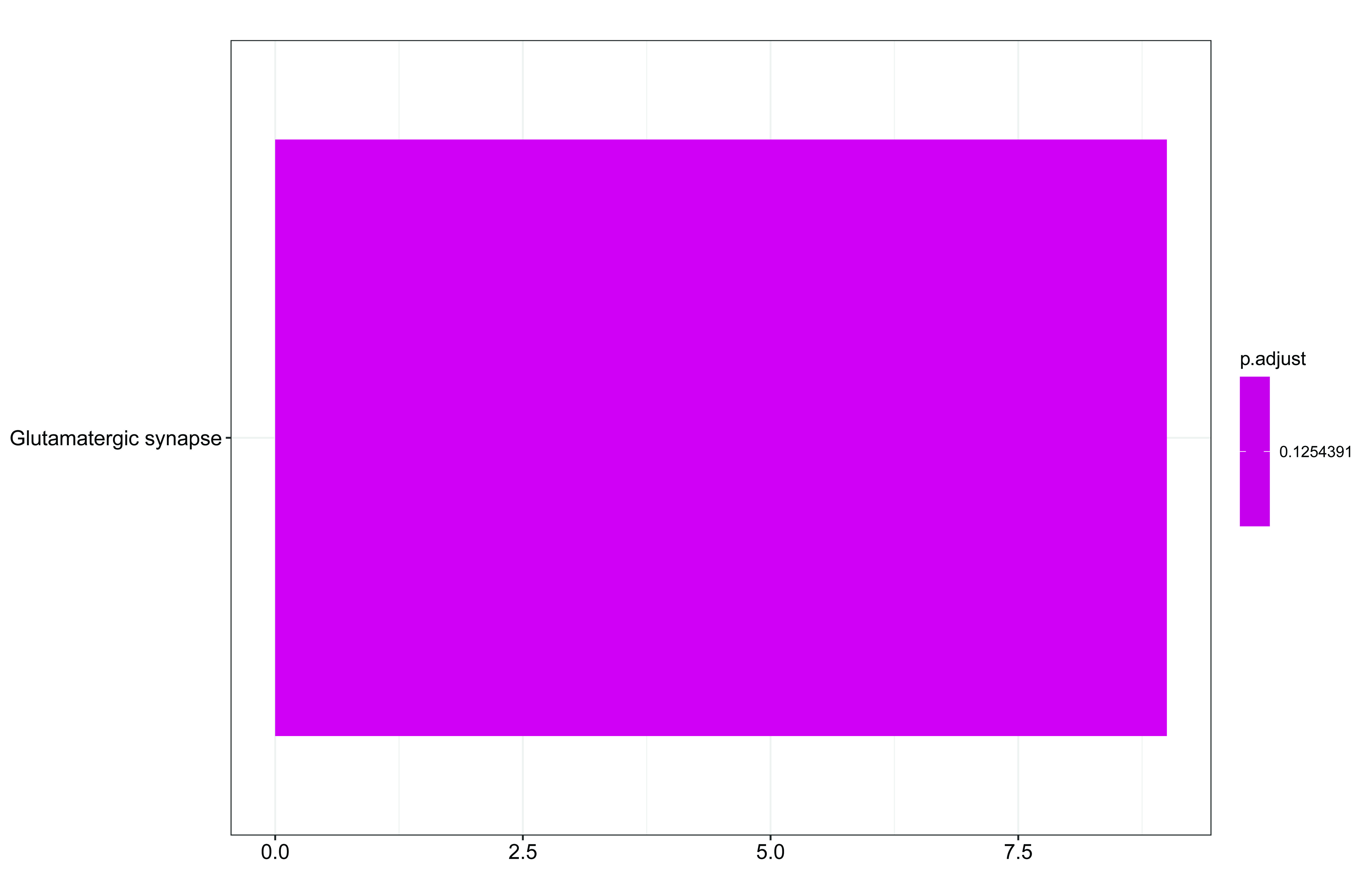

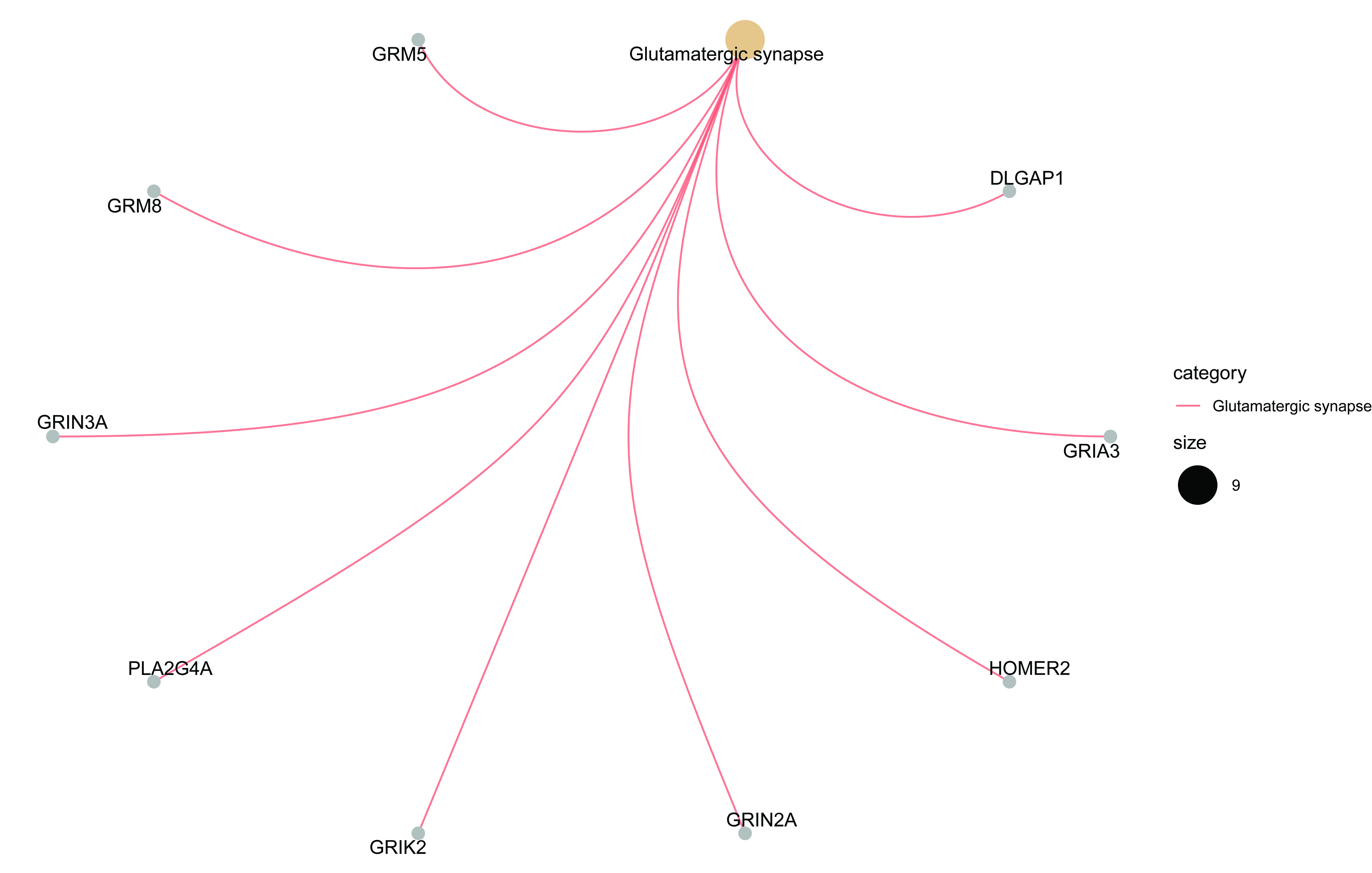


**Figure S7. Enrichments of HBV integration breakpoints in KEGG pathways were found in patients with lower inflammation activity grade score in liver histology.** (A, C) Histogram of HBV integration breakpoints in KEGG pathways. The vertical and horizontal axes illustrate the name of pathway and the count of the enriched genes, respectively. The color shows the statistical significance with red relating to a smaller *P*-value. (B, D) Network map of the pathways and their HBV integration-related genes. Gray node in the figure represents a gene; Orange node represents the pathway and the circle size represented gene numbers in the pathway; Color line between the nodes represented the connection of pathways and genes.


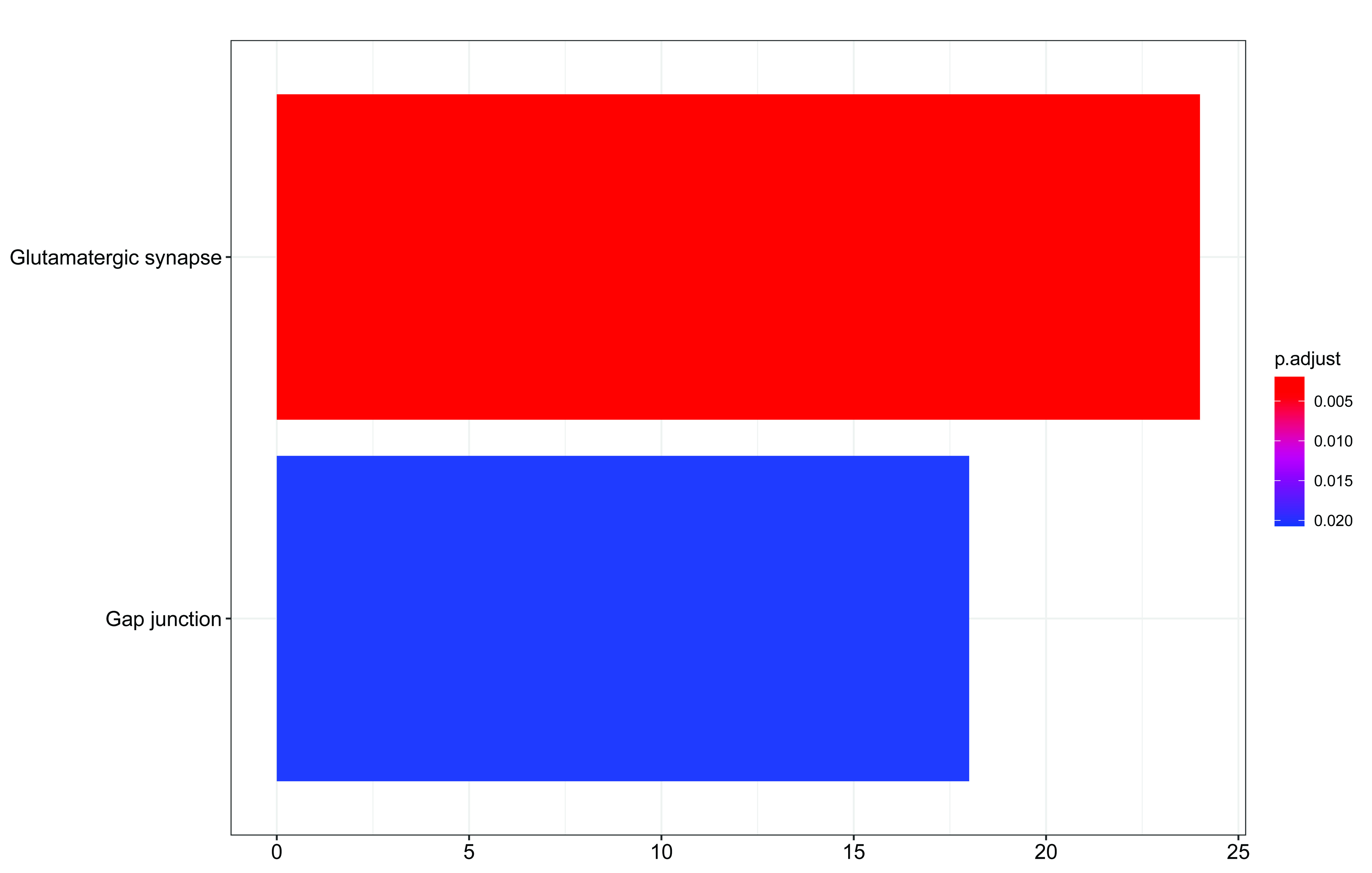

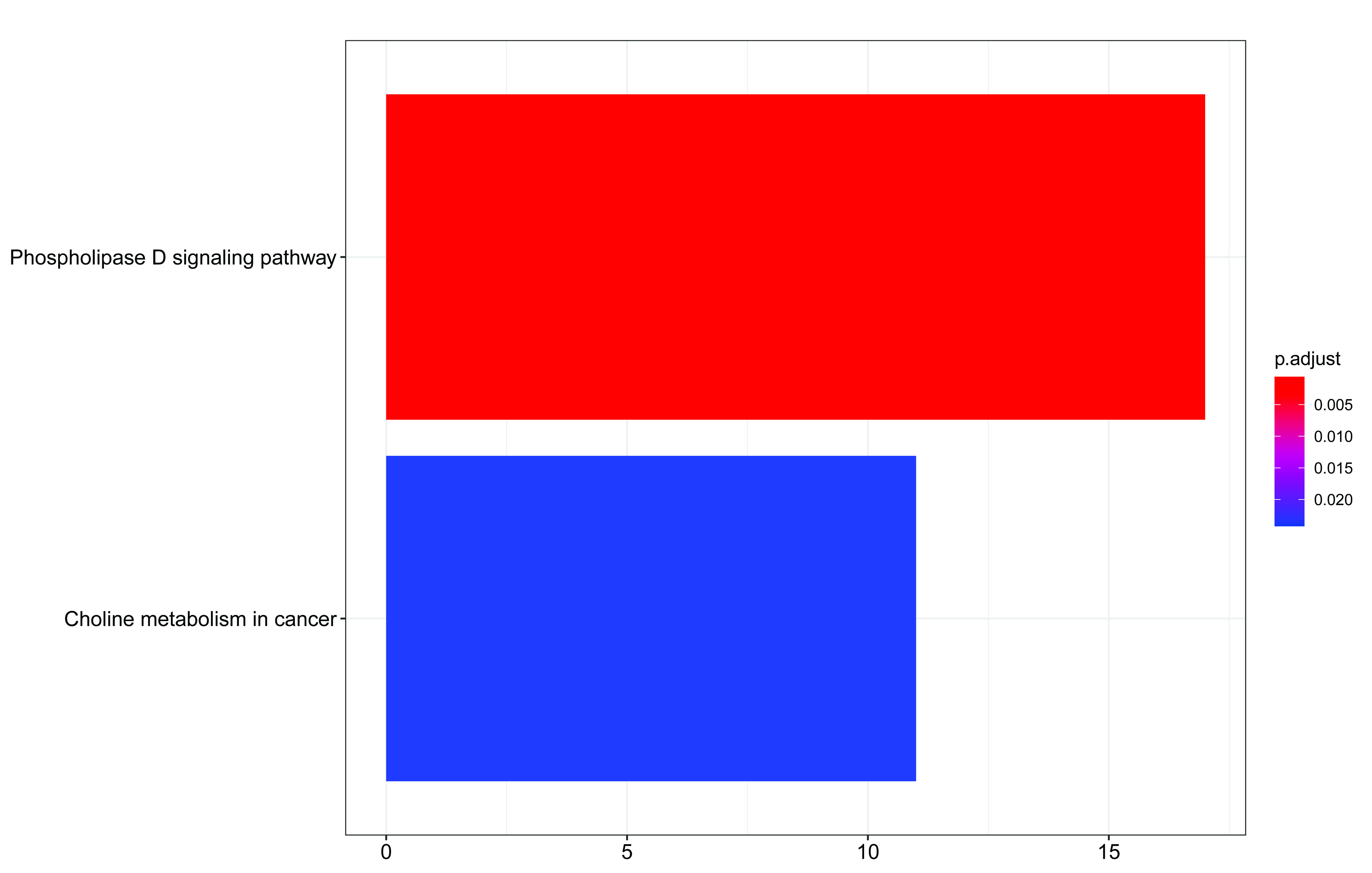

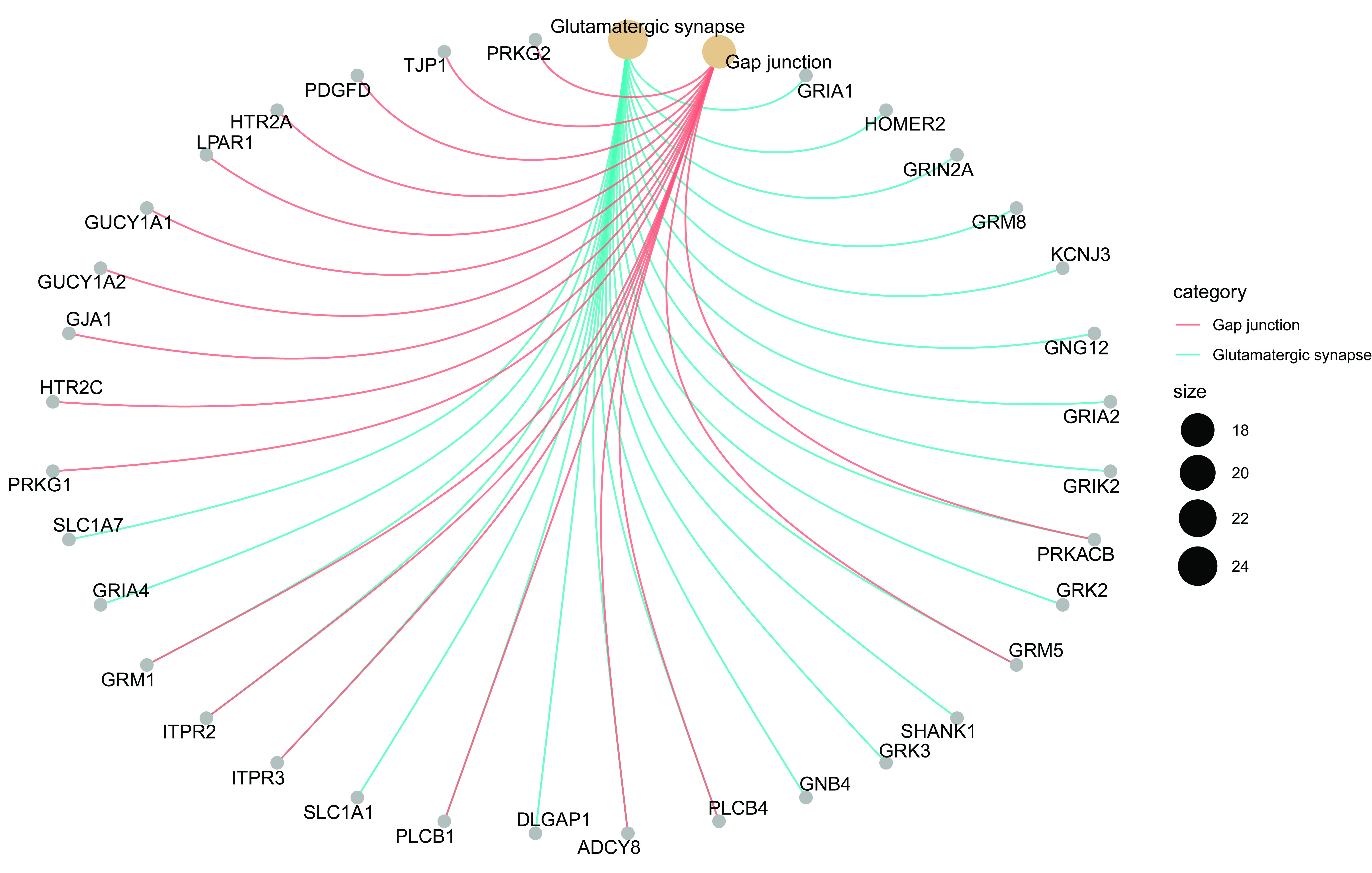

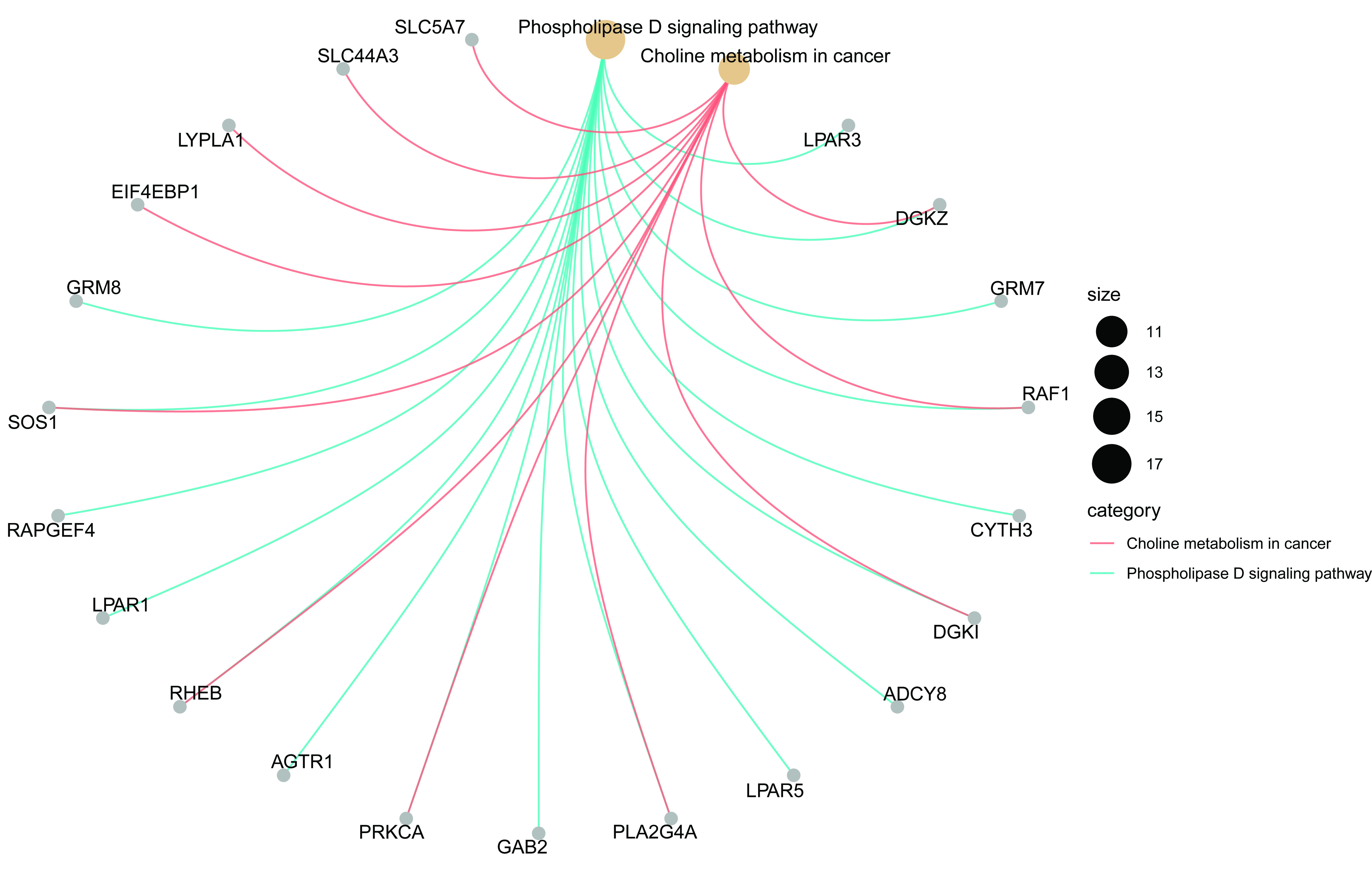


**Figure S8. Enrichments of HBV integration breakpoints in KEGG pathways were different in patients with high and low fibrosis scores in liver histology.** (A, C) Histogram of HBV integration breakpoints in KEGG pathways. The vertical and horizontal axes illustrate the name of pathway and the count of the enriched genes, respectively. The color shows the statistical significance with red relating to a smaller *P*-value. (B, D) Network map of the pathways and their HBV integration-related genes. Gray node in the figure represents a gene; Orange node represents the pathway and the circle size represented gene numbers in the pathway; Color line between the nodes represented the connection of pathways and genes.


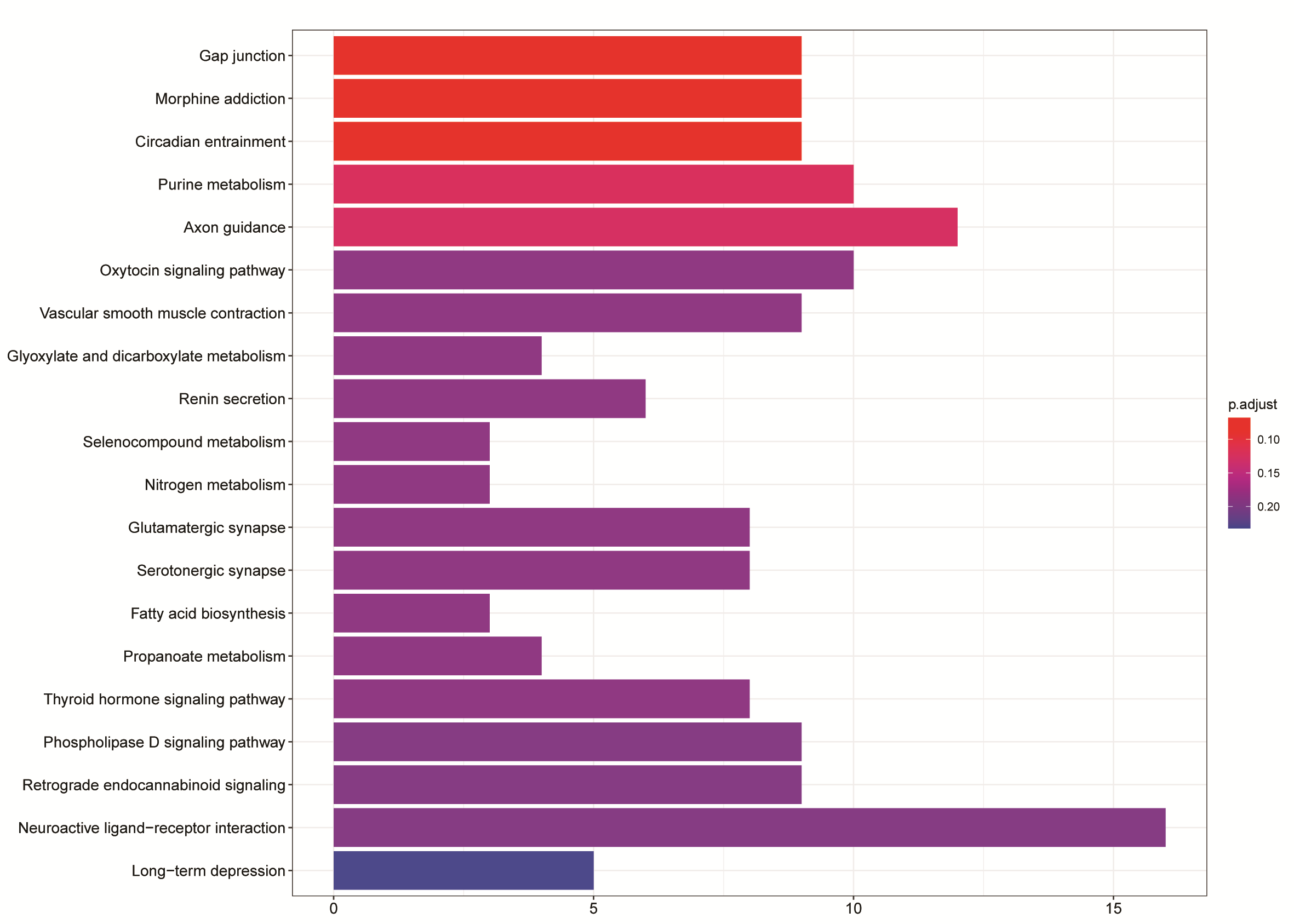

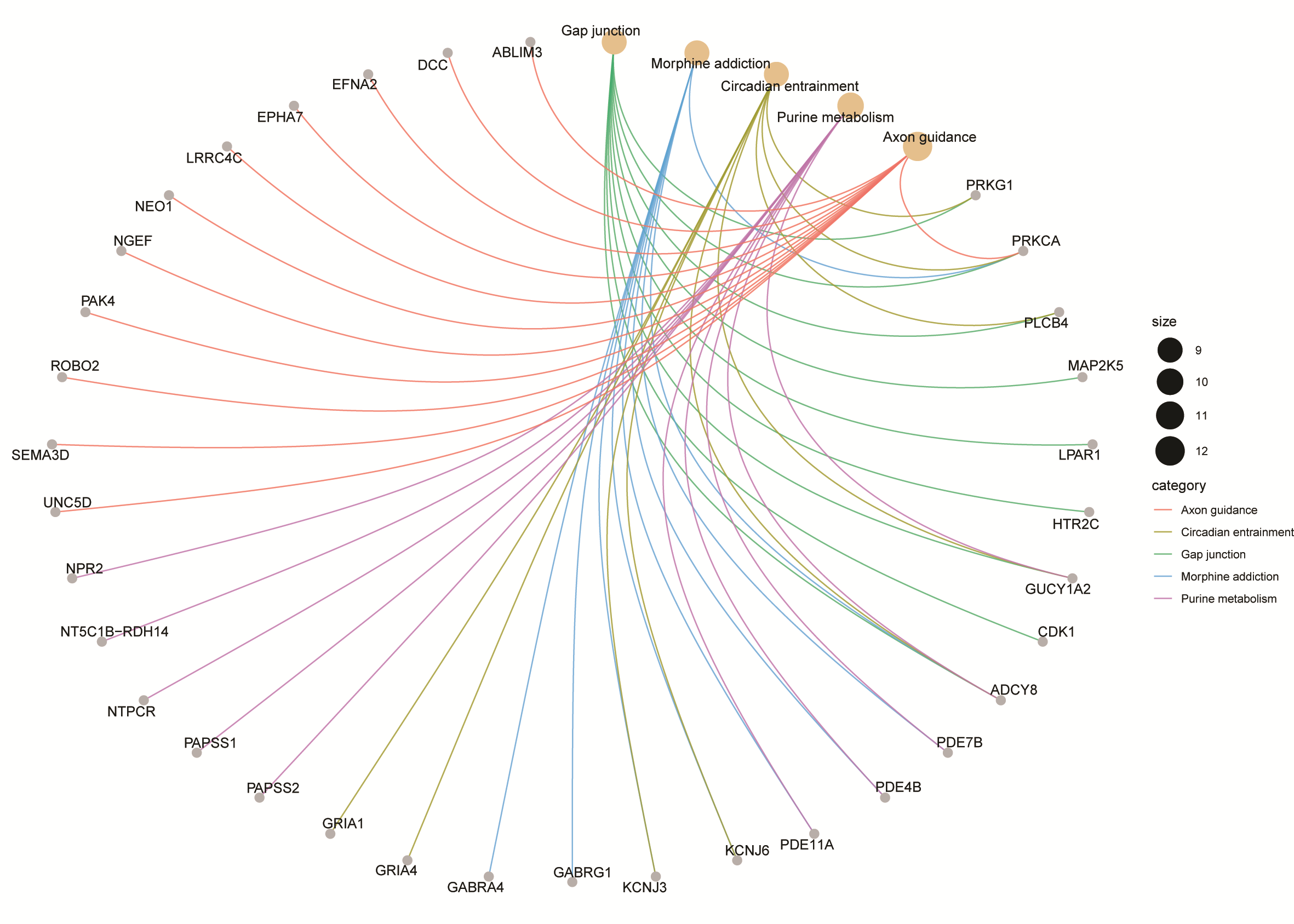


**Figure S9. No enrichments of HBV integration breakpoints in KEGG pathways in NUC-treated patients.** (A) Histogram of HBV integration breakpoints in KEGG pathways. The vertical and horizontal axes illustrate the name of pathway and the count of the enriched genes, respectively. The color shows the statistical significance with red relating to a smaller *P*-value. (B) Network map of the pathways and their HBV integration-related genes. Gray node in the figure represents a gene; Orange node represents the pathway and the circle size represented gene numbers in the pathway; Color line between the nodes represented the connection of pathways and genes.


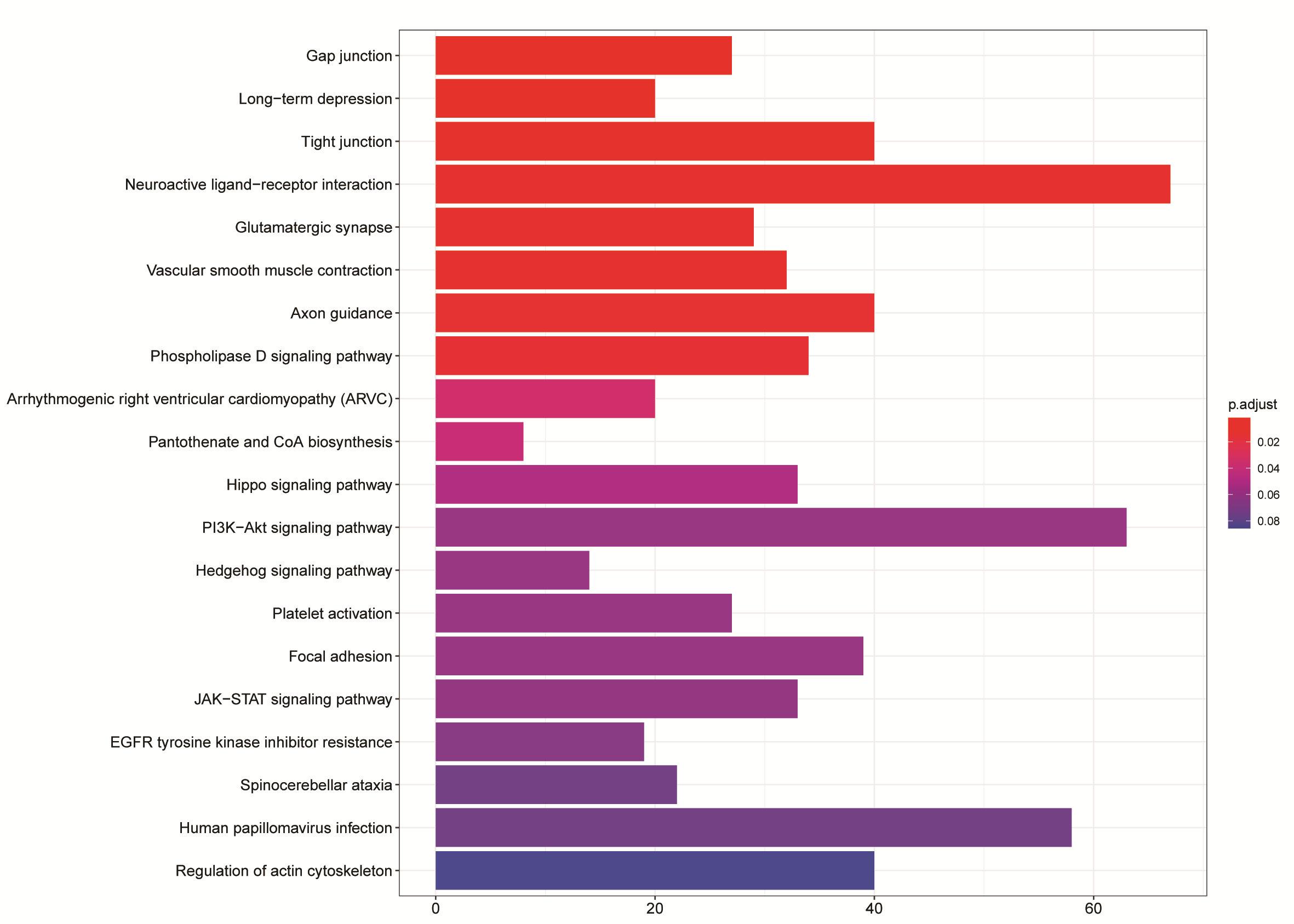

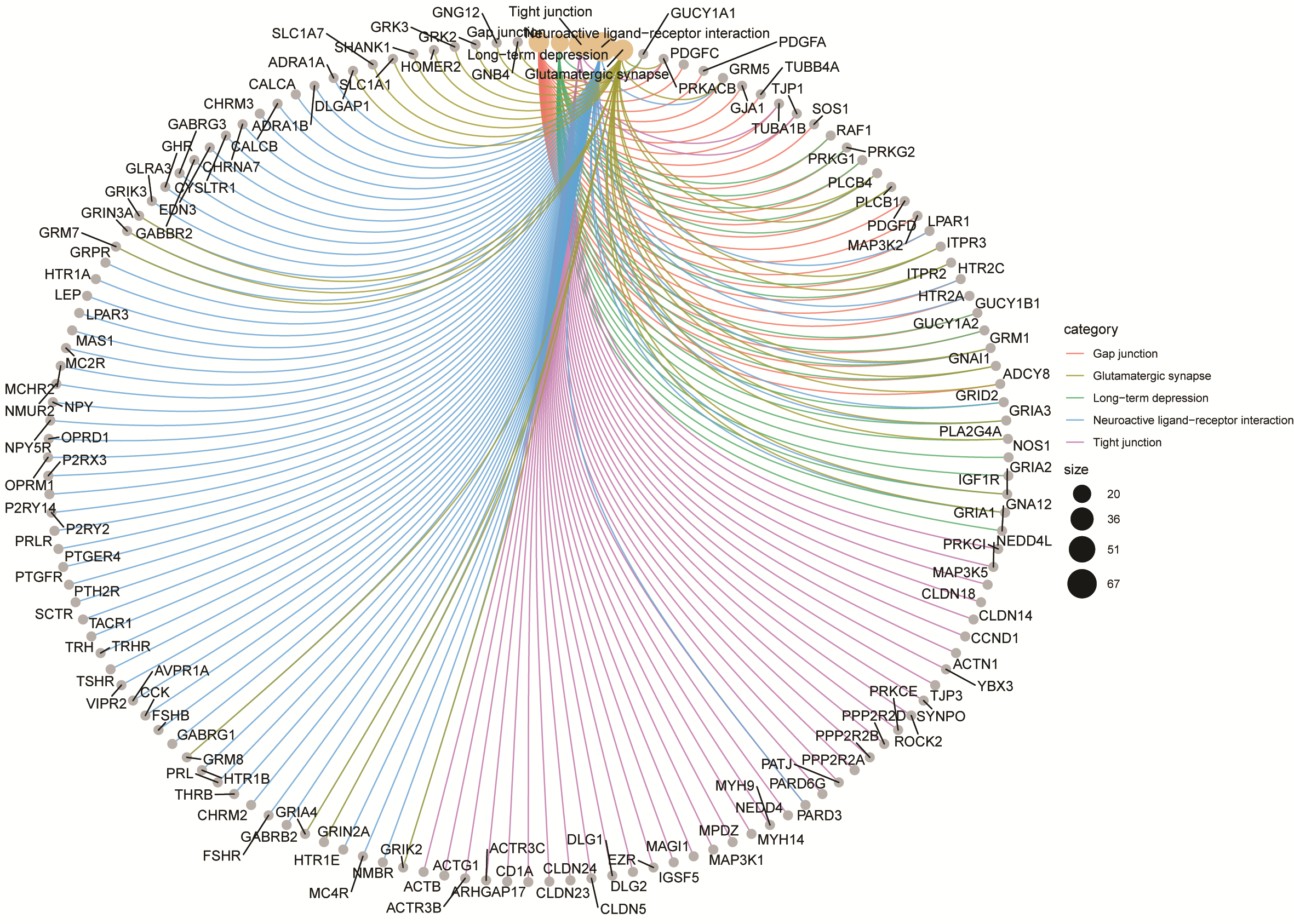


**Figure S10. Enrichments of HBV integration breakpoints in KEGG pathways in non-NUC-treated patients.** (A) Histogram of HBV integration breakpoints in KEGG pathways. The vertical and horizontal axes illustrate the name of pathway and the count of the enriched genes, respectively. The color shows the statistical significance with red relating to a smaller *P*-value. (B) Network map of the pathways and their HBV integration-related genes. Gray node in the figure represents a gene; Orange node represents the pathway and the circle size represented gene numbers in the pathway; Color line between the nodes represented the connection of pathways and genes.
